# Alternative Splicing of *SORBS1* Affects Neuromuscular Junction Integrity in Myotonic Dystrophy Type 1

**DOI:** 10.1002/jcsm.70112

**Published:** 2025-11-18

**Authors:** Caroline Hermitte, Hortense de Calbiac, Gilles Moulay, Antoine Mérien, Jeanne Lainé, Hélène Polvèche, Michel Cailleret, Stéphane Vassilopoulos, Edor Kabashi, Denis Furling, Cécile Martinat, Morgan Gazzola

**Affiliations:** ^1^ INSERM/UEPS UMR 861, Paris Saclay University, I‐STEM Corbeil‐Essonnes France; ^2^ Laboratory of Translational Research for Neurological Disorders Imagine Institute, Université de Paris, INSERM UMR 1163 Paris France; ^3^ Centre de Recherche en Myologie Sorbonne Université, INSERM, Institut de Myologie Paris France; ^4^ CECS/AFM, I‐STEM Corbeil‐Essonnes France

**Keywords:** alternative splicing, myotonic dystrophy type 1, neuromuscular junction

## Abstract

**Background:**

Myotonic dystrophy type 1 (DM1) is a multisystemic neuromuscular disorder characterized by CTG repeat expansion in the 3′ untranslated region of the *dystrophia myotonica protein kinase* coding gene. The presence of expanded CTG repeats in DMPK mRNAs leads to the sequestration of RNA‐binding factors such as the Muscleblind‐like (MBNL) proteins, resulting in widespread splicing defects that contribute to progressive muscle weakness and myotonia. Previously, we identified misregulation of *SORBS1* exon 25 splicing in both DM1 and MBNL1/2 double‐knockout human‐induced pluripotent stem cells (hiPSC)‐derived skeletal muscle cells, suggesting a potential role in DM1 physiopathology.

**Methods:**

We investigated *SORBS1* exon 25 splicing misregulation in human skeletal muscle biopsies from DM1 patients and healthy controls. The functional consequence of *SORBS1* exon 25 exclusion was assessed in mice, zebrafish and hiPSC‐derived skeletal muscle cells using an antisense oligonucleotide‐mediated exon‐skipping strategy.

**Results:**

In human congenital DM1 fetal skeletal muscle biopsies, *SORBS1* exon 25 inclusion was reduced by 52.6 ± 10% compared to controls (*p < 0.001*). Analysis of RNA sequencing data from the DMseq database further revealed significant misregulation in tibialis anterior biopsies from 40 adult DM1 patients, with a 15.8 ± 3.7% decrease in splice inclusion (*p < 0.0001*). In mice, forced exclusion of *Sorbs1* exon 25 led to neuromuscular junction degeneration, with increased denervation (10.5% ± 3.4%, *p < 0.01*) and postsynaptic destabilization (5.7% ± 2.5%, *p < 0.05*). In zebrafish, *sorbs1* exon 25 misregulation significantly impaired locomotion, reducing trajectory, distance (57.9% ± 12%, *p < 0.0001*) and velocity (14% ± 0.5%, *p < 0.05*), while also disrupting acetylcholine receptor cluster morphology. Similarly, forced *SORBS1* exon 25 exclusion in hiPSC‐derived skeletal muscle cells diminished the formation of large acetylcholine receptor clusters upon agrin stimulation by 34% *±* 4.5% (*p < 0.0001*).

**Conclusion:**

Our study identifies *SORBS1* alternative splicing as an essential MBNL‐regulated event during skeletal muscle development, potentially involved in neuromuscular junction formation and maintenance. The aberrant splicing of *SORBS1* exon 25 in DM1 expands our understanding of how splicing dysregulation compromises neuromuscular system communication, shedding light on the broader impact of mRNA splicing regulation on NMJ integrity.

## 1 Introduction

Myotonic dystrophy type 1 (DM1), with a prevalence of 1 over 8000 individuals, is considered one of the most common neuromuscular disorders worldwide [1]. This pathology is characterized by muscular alterations such as myotonia, muscle atrophy and muscle weakness [2]. DM1 is caused by the presence of CTG repeat expansions in the 3′ untranslated region (UTR) of the gene coding for the *dystrophia myotonica protein kinase* (DMPK). The severity of this disease correlates with the number of CTG repeats, ranging from congenital forms (> 1500 repetitions) to adult forms and late onset (50–150 repetitions). Once transcribed, the CUG repeats form hairpin structures that sequester RNA‐binding factors such as Muscleblind‐like (MBNL) proteins, resulting in the formation of toxic foci within the cell nuclei [3].

The sequestration of MBNL proteins results in widespread misregulation of alternative splicing, including cassette exon, mutually exclusive exon and retained introns [4]. To date, only a few mis‐splicing events affecting genes such as *CLCN1*, *CLTC*, *BIN1* and *DMD* have been characterized at the pathological level and shown to contribute to muscle weakness and myotonia [5, 6, 7]. Additional splicing misregulation events have been identified in DM1, but their impact on muscle function remains largely unexplored [8]. Among them, misregulation of *SORBS1* exon 25 splicing has also been reported in DM1 mouse models, including DMSXL and MBNL1 deficient mice [9, 10]. We recently identified *SORBS1* exon 25 as one of the most significantly differentially spliced exons (DSEs) in DM1‐affected human‐induced pluripotent stem cells (hiPSC)‐derived skeletal muscle cells, and in cells lacking MBNL isoforms 1 and 2 of MBNL (MBNL DKO) [11].

The *SORBS1* gene encodes a protein involved in cytoskeleton modulation through interactions with paxillin, talin and vinculin [12, 13] and has recently been described as implicated in acetylcholine receptor (AChR) cluster formation in mouse skeletal muscle cells [14]. These observations suggest that *SORBS1* splicing misregulation may play a previously unrecognized role in NMJ formation and stability. Given the critical role of NMJs in muscle contractions and locomotion, we hypothesized that mis‐splicing of *SORBS1* exon 25 could significantly impair NMJ formation and maintenance in DM1. To explore this, the impact of *SORBS1* exon 25 splicing misregulation on NMJ integrity was investigated.

By utilizing forced exon‐skipping strategies across diverse in vitro and in vivo models, including hiPSC‐derived skeletal muscle cells, mice and zebrafish, our findings demonstrate that the SORBS1 protein encoded from the transcript containing exon 25 may play a crucial role in NMJ formation and stability. Furthermore, the misregulation of *SORBS1* exon 25 alternative splicing could contribute to pathological motor impairments.

## 2 Materials and Methods

### 2.1 Human Cells and Tissue Samples

Fetal skeletal muscle samples were obtained from autopsies, in accordance with the French legislation on ethical rules. As previously described [7], control and congenital DM1 muscle samples were obtained from aborted fetuses showing, respectively, no sign of neuromuscular disease (control) and clinical symptoms of congenital DM1 form with large CTGn > 1000 repeats.

Human primary skeletal muscle cells (provided by Dr. Furling, Institute of Myology, Paris) were isolated from a muscle biopsy of a healthy child obtained via Myobank‐AFM, affiliated with EuroBioBank, in accordance with European recommendations and French legislation (authorization AC‐2019‐3502). Myoblasts were thawed and cultivated in a growth medium consisting of DMEM‐F12 glutamax (Life Technologies) supplemented with 20% FBS (Sigma‐Aldrich), 1% Insulin human solution (Sigma‐Aldrich) and 0.1% penicillin/streptomycine (Life Technologies). Once the cells reached confluence, myogenic differentiation was induced by switching the cell cultures to DMEM supplemented with 2% horse serum (Life Technologies) for 3 days to allow myotube formation.

### 2.2 Bioinformatic Analysis

RNA‐seq data from GSE86356, Tibialis anterior muscle tissue [15] were analysed using Human Genome Reference GRCh37.87, STAR aligner (Version 2.7.3a) and the Multivariate Analysis of Transcript Splicing (rMATS Version 4.1.2) program to identify alternatively skipped exons (ASE—JC count) between myotonic dystrophy type 1 (DM1) patients and control. Spliced exon variants, once detected, are significant when they have an adjusted *p*‐value ≤ 0.05 and a ΔPSI value ≥ 10%.

### 2.3 AAV Production and Titration

U7snRNA antisense sequences were cloned into pSMD2‐U7‐BsmI to target SORBS1 exon 25 skipping. AAV1 or AAV9 viral vectors were produced via tri‐transfection in 293 cells. Vector particles were purified on iodixanol gradient and concentrated on Amicon Ultra‐15 100K columns (Merck‐Millipore, USA). The AAV vectors were titrated as viral genomes (vg) per millilitre by quantitative real‐time PCR purified on iodixanol gradients and titrated by qPCR using ITR2 primers.

### 2.4 Mouse Experiments

Studies adhered to French ethical laws (approval #13333‐2018013111391590 v2). Eight‐week‐old female FVB mice received intramuscular AAV‐U7‐SORBS1‐ESE25 injections into the left tibialis anterior (TA), with contralateral PBS controls. Dose–response studies (0.5–8 × 10^11^ vg) identified optimal doses at 4 × 10^11^ vg (AAV1) and 8 × 10^11^ vg (AAV9). Mice were sacrificed at 2 or 6 months post‐injection, with the collection of TA muscles prepared for either electron microscopy, RNA extraction or neuromuscular junction immunofluorescence.

For transmission electron microscopy, TA muscles were fixed, postfixed, stained with uranyl acetate, embedded in epoxy resin, sectioned and imaged via JEOL TEM.

For NMJ immunofluorescence, fibres were stained with neurofilament (DSHB Hybridoma Product 2H3), synaptophysin (Invitrogen #PA527286), α‐bungarotoxin (Invitrogen #B13432) and Alexa Fluor‐conjugated secondary antibodies, then imaged via Nikon Ti2 spinning disk confocal microscopy. Images were acquired as Z‐stacks, and a semi‐automated morphometric analysis was performed using the ImageJ software (https://imagej.nih.gov/ij/) and the NMJ‐morph macro to quantify nerve terminal, endplate and the acetylcholine receptor areas.

### 2.5 RNA Extraction and RT‐PCR

Total RNA was isolated using TRIZOL reagent (Life Technologies Cat#15596018, France) from mice TA muscle, human samples or hiPS cell cultures. Total RNA (1 μg) was submitted to reverse transcription using MLV reverse transcriptase and oligo dT_12–18_ (Life Technologies, France). PCR was performed using 25 ng of cDNA diluted in platinum Taq polymerase mix. The different primers used in this study, both for RT‐PCR and for RT‐qPCR, are listed in Table S1. Gel electrophoresis of PCR products was performed in 2.5% agarose, and images were acquired on a Geni2 gel imaging system (Ozyme, France). The densitometric analysis of PCR bands was realized using ImageJ Software.

### 2.6 RT‐qPCR

Quantitative PCR was performed using Luna Universal qPCR Master Mix (New England Biolabs) on a QuantStudio 12K. Each reaction was performed in technical triplicates, and gene expression was normalized using the ΔΔCt method.

### 2.7 Zebrafish Experiments

Adult and larval zebrafish ( *Danio rerio* ) were maintained at the Imagine Institute (Paris) facility and bred according to the National and European Guidelines for Animal Welfare. Experiments were performed on wild‐type zebrafish larvae from AB strains. Zebrafish were staged in terms of hours post fertilization (hpf) based on morphological criteria and manually dechorionated using fine forceps at 24 hpf. All the experiments were conducted on morphologically normal zebrafish larvae.

A morpholino antisense oligonucleotide (MO; GeneTools, Philomath, USA) was used to specifically knock down the expression of the *sorbs1* zebrafish orthologue. The MO was designed to bind to a splicing region in exon 25 (sorbs1‐MO). The sequence is 5′‐ACA‐AGA‐GAG‐AAC‐AUU‐CAU‐CAC‐CUC‐U‐3′. Control morpholino (control‐MO), not binding anywhere in the zebrafish genome, has the following sequence 5′‐CCT‐CTT‐ACC‐TCA‐GTT‐ACA‐ATT‐TAT‐A‐3′. MOs were injected at the final concentration of 0.6 mM.

Locomotor behaviour of 50 hpf zebrafish larvae was assessed using the Touched‐Evoked Escape Response (TEER) test. Travelled distance and average velocity were quantified frame by frame for each embryo using the video tracking plugin of FIJI 1.83 software. All phenotypic and quantitative analyses were conducted exclusively on morpholino‐injected embryos displaying normal overall morphology without overt developmental abnormalities.

### 2.8 Cell Lines

The hiPSC lines used in this study have been previously described [11]. Briefly, DM1 hiPS cell lines (derived from male donors, with > 2000 CTG repeats), control hiPS cells and *MBNL1,2* KOs hiPSCs (both derived from female donors) were maintained and passaged in StemMACS iPS‐Brew XF medium (Miltenyi Biotec) in vitronectin‐coated culture dishes (Gibco). Skeletal muscle differentiation experiments were performed using the commercially available STEMdiff Myogenic Progenitor Supplement Kit (StemCell Technologies). For terminal differentiation, cells were thawed in MyoCult‐SF Expansion Supplement Kit (StemCell Technologies) for 2–3 days. When the cells reached confluence, the medium was replaced with MyoCult Differentiation Kit (StemCell Technologies), and the cells were maintained in culture for 7 days to allow myotube formation. RNA‐Seq: Data are available on the GEO database (GSE161897).

### 2.9 ASO(s) Treatments

Differentiated hiPSCs‐derived control myotubes or human primary control myoblasts were transfected at Day 5 of differentiation with 50 nM 2‐OMe ASOs using RNAi Max transfection reagent (Life Technologies) according to the manufacturer's protocol. At 48 h post‐transfection, cells were stimulated with 0.5 μg/mL rat agrin (R&D Systems) for various time periods depending on the experiment and harvested for analysis.

### 2.10 Lentivirus Infection

Proliferating DM1 hiPSC‐derived myoblasts were infected for 24 h in DMEM high glucose (#36250 Stem Cell Tech) in the presence of polybrene at 5 μg/mL and subsequently allowed to recover for an additional 24 h. hiPSC‐derived myoblasts were transduced at a multiplicity of infection (MOI) ranging from 0.5 to 5. The viral dose was calculated based on the number of cells per well for each condition. The cells were subsequently differentiated into myotubes over a 7‐day period and stimulated with 0.5 μg/mL agrin for 6 h. AChR cluster size was then quantified by confocal microscopy. Notably, we observed a modest increase in the mean AChR cluster size in DM1‐derived myotubes following transduction at an MOI of 1.

### 2.11 Immunocytochemistry

Cells were fixed for 10″ in PBS containing 4% PFA and 0.1% Triton X100. Cells were washed three times with PBS and blocked for 1 h in PBS containing 3% BSA and 0.1% Tween20. Primary antibodies were incubated for 30″ at RT in PBS containing 3% BSA and 0.1% Tween20. Cells were washed three times in PBS‐Tween20 0.1% and incubated for 30″ at RT with appropriate fluorescently labelled secondary antibodies. Images were acquired using two different microscopes. Zebrafish sections and hiPSC‐derived myotubes for AChR cluster quantification were imaged using a confocal spinning disk microscope (Zeiss Axio Observer Z.1). Images were acquired using a Plan‐Apo 63x/1.40 NA oil objective or a Plan‐Apo 20x/0.8 NA air objective. High‐magnification images were obtained on a scanning confocal microscope (Zeiss LSM 880) using Airyscan detector mode.

### 2.12 Western Blot and Co‐IP

Proteins were extracted from cell lysates using RIPA buffer supplemented with protease and phosphatase inhibitors. Protein concentration was quantified using the BCA assay (Thermo Fisher Scientific). Twenty micrograms of total protein per sample were denatured in Laemmli buffer and resolved on SDS‐PAGE gels, followed by transfer to PVDF membranes using a semi‐dry (iBlot2) transfer system. Membranes were blocked with 5% non‐fat dry milk in TBS‐T (Tris‐buffered saline with 0.1% Tween‐20) for 1 h at room temperature and incubated overnight at 4°C with primary antibodies diluted in blocking solution. After washing, membranes were incubated with HRP‐conjugated secondary antibodies for 1 h at room temperature. Signal detection was performed using enhanced chemiluminescence (Amersham) and visualized using a chemiluminescence imaging system. Band intensities were quantified using ImageJ, and protein levels were normalized to β‐actin.

Co‐immunoprecipitation was performed using 100 μg of total protein from cell lysates prepared in NP‐40 containing lysis buffer supplemented with protease and phosphatase inhibitors. Lysates were pre‐cleared with protein G Sepharose beads (ab193259) for 1 h at 4°C to reduce nonspecific binding. For immunoprecipitation, 1 μg of the polyclonal CRKL antibody (PA5‐28622) was incubated with the lysate for 2 h at 4°C with gentle rotation. The immune complexes were captured by incubation with 70 μL of pre‐washed protein G Sepharose beads for 1 h at 4°C. Beads were then washed with lysis buffer, and bound proteins were eluted by boiling samples in Laemmli buffer. Eluates were analysed by SDS‐PAGE followed by Western blotting using specific SORBS1 antibodies to detect co‐precipitated proteins.

### 2.13 Statistical Analysis

All data were processed using Prism 9. For statistical analysis, either Student's *t*‐test or one‐way analyses of variance were used as appropriate. Values are represented as mean ± SD Values of *p* < 0.05 were considered significant (*p < 0.05; **p < 0.01; ***p < 0.001; ****p < 0.0001).

## 3 Results

### 3.1 *SORBS1* Exon 25 Is Misregulated in Skeletal Muscle From DM1 Patients

**FIGURE 1:**
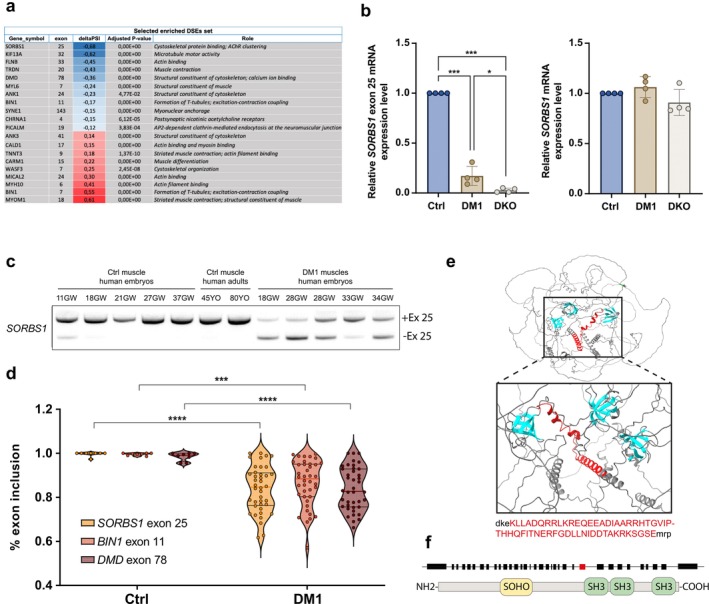
*SORBS1* exon 25 is misregulated in skeletal muscle biopsies from DM1 patients. (a) List of 30 enriched DSEs identified in common in DM1, DKO MBNL1/2 and WT hiPSC‐derived skeletal muscle cells in bulk transcriptomic analysis. For alternative splicing analysis, exons have been numbered according to FasterDB database. (b) RT‐qPCR analysis of *SORBS1* exon 25 inclusion and total *SORBS1* mRNA in hiPSC‐derived skeletal muscle cells from WT, DM1 and DKO MBNL1/2. (*N* = 4 independent experiments, ****p < 0.0001; ***p < 0.001; *p < 0.01, one‐way ANOVA followed by Tukey's post hoc test). (c) RT‐PCR analysis of *SORBS1* exon 25 alternative splicing in human skeletal muscle samples from control (CTL) and congenital DM1 fetuses (DM1). (d) Violin plots of *SORBS1* exon 25, *BIN1* exon 11 and *DMD* exon 78 inclusion in tibialis anterior muscles samples from control and DM1 patients obtained from the publicly available DMseq database. Ctrl (*N* = 11); DM1 (*N* = 40). (****p < 0.00001; ***p < 0.0001, one‐way ANOVA followed by Tukey's post hoc test). (e) AlphaFold analysis of the SORBS1 tridimensional structure. The sequences highlighted in red correspond to the exon 25. (f) Schematic diagram of the SoHo domain and the three SH3 domains of the SORBS1 protein. Black squares represent coding exons, and the red box corresponds to the exon 25.

In our previous study, we identified widespread alternative splicing misregulation in both DM1 and MBNL1/2 double knockout (DKO) hiPSC‐derived skeletal muscle cells [11]. This analysis revealed 254 DSEs across control, DM1 and DKO conditions. Notably, *SORBS1* exon 25 exhibited the most significant downregulation among these DSEs, with a marked reduction in the percentage of splice inclusion (ΔPSI) of 68% in DM1 myotubes compared to controls (Figure 1a). RT‐qPCR analyses performed in control, DM1 and DKO hiPSC‐derived skeletal muscle cells corroborated this finding, with a ΔPSI of 82.8 ± 3.9% in DM1 skeletal muscle cells and an even more pronounced reduction of 97.2 ± 2.6% in DKO skeletal muscle cells, confirming the regulatory influence of MBNL1/2 proteins on *SORBS1* splicing (Figures 1b and S1a). These splicing changes occurred without any significant alteration in total *SORBS1* expression levels (Figure 1b).

To assess whether *SORBS1* exon 25 undergoes developmental regulation in human skeletal muscles and whether it is misregulated in DM1 patients, exon 25 inclusion was measured in skeletal muscle samples from control and DM1 fetuses. In control samples, the inclusion of *SORBS1* exon 25 progressively increased during development, with full inclusion after 11 gestational weeks (GW), highlighting its significance during early skeletal muscle formation (Figure 1c). In sharp contrast, *SORBS1* exon 25 remained misregulated throughout skeletal muscle development in congenital DM1 fetuses, failing to reach full inclusion even at 28 and 34 GW (Figure 1c). The ΔPSI for exon 25 in congenital DM1 fetal skeletal muscles was significantly reduced, with a mean value of 52.6 ± 10% (Figure S1b). This finding underscores a persistent developmental defect in splicing regulation in DM1.

To confirm the misregulation of *SORBS1* exon 25, the analysis was extended to a larger cohort of DM1 patient samples. Using publicly available RNA sequencing data from the DMseq database (http://www.dmseq.org), and consistent with our initial findings, *SORBS1* exon 25 exhibited significant misregulation across *tibialis anterior* (TA) muscle biopsies from adult DM1 patients with a ΔPSI of 15.8 ± 3.7% without affecting total transcript expression (Figures 1d and S1c). This level of misregulation was comparable to that observed for *BIN1* exon 11 (13.1 ± 3.7%) and *DMD* exon 78 (14.5 ± 3.5%), two well‐characterized MBNL regulated alternative splices and known to be deregulated in DM1 (Figure 1d) [6, 7].

### 3.2 Splicing Misregulation of *SORBS1* Exon 25 Impairs Mice NMJ Stability

**FIGURE 2:**
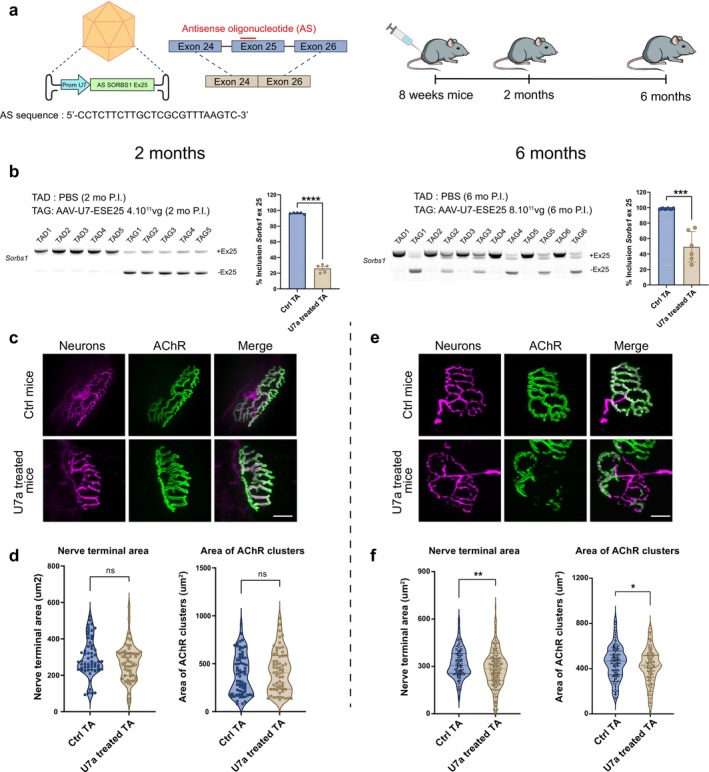
Exclusion of *Sorbs1* exon 25 in mice skeletal muscle affects NMJ stability. (a) Schematic representation of the U7—*Sorbs1* exon 25 antisense constructs (U7‐ESE25) and the experimental protocol. (b) RT‐PCR analysis and quantification of *Sorbs1* exon 25 inclusion in TA muscles injected with AAV‐U7‐ESE25 compared with contralateral TA muscles injected with PBS (Ctrl) 2 months (*p* < 0.0001, *N* = 5 mice, Student's *t*‐test) and 6 months post injection (*p* < 0.001, *N* = 5 mice, unpaired Student's *t*‐test). (c) Representative immunofluorescence from confocal Z projections of NMJ from TA muscles injected with AAV‐U7‐ESE25 compared with contralateral TA muscles injected with PBS (Ctrl) 2 months post injection. (d) Violin plots of nerve terminal area and AChR area output using NMJ‐Morph. Ctrl TA (*n* = 57) from *N* = 5 mice; U7‐treated TA (*n* = 54) from *N* = 5 mice. (e) Representative immunofluorescence from confocal Z projections of NMJ from TA muscles injected with AAV‐U7‐ESE25 compared with contralateral TA muscles injected with PBS (Ctrl) 6 months post injection. (f) Violin plots of nerve terminal area and AChR area output using NMJ‐Morph. Ctrl TA (*n* = 234) from *N* = 6 mice; U7‐treated TA (*n* = 327) from *N* = 6 mice. *p* < 0.01 and *p* < 0.05, unpaired Student's *t*‐test.

To investigate the potential role of *SORBS1* exon 25 inclusion, *in silico* modelling was initially performed with AlphaFold2 [16] to predict its localization and possible impact on the protein structure (Figure 1e). The predicted structure showed that exon 25 codes for two α‐helices, located just upstream of the first SRC homology (SH3) domain, suggesting that this region could be critical for the functional conformation of the SORBS1 protein (Figure 1e–f). To investigate the physiological consequences of *SORBS1* exon 25 mis‐splicing in DM1, we employed an exon‐skipping strategy. We designed antisense oligonucleotides (ASOs) to target specific sequences in *SORBS1* pre‐mRNA and promote exon 25 exclusion (Figure S2). ASO efficacies were assessed by quantifying exon 25 inclusion 48 h post‐transfection in human primary skeletal muscle cells (Figure S2b). Among the different ASOs tested, only one successfully induced an exclusion level comparable to that observed in DM1 skeletal muscle cells by targeting a putative exonic splicing enhancer (ESE25). To determine whether ASO treatment influenced overall *SORBS1* expression level, we measured the total *Sorbs1* mRNA levels by quantitative PCR (qPCR) (Figure S2c–d). Interestingly, ASO‐mediated exon 25 exclusion did not alter total *SORBS1* mRNA expression but selectively reduced the number of transcripts containing exon 25 (Figure S2d). To note, ASO‐mediated exon 25 exclusion did not alter SORBS2 mRNA expression level, another member of the SORBS gene family [13] (Figure S2d). We then engineered a modified U7 small nuclear RNA (U7‐snRNA) construct harbouring the antisense sequence targeting ESE25 (*U7‐SORBS1‐ESE25*) and cloned it into an adeno‐associated virus (AAV) (Figure 2a). The left TA muscles of wild‐type eight‐week‐old female mice were then injected with *U7‐SORBS1‐ESE25* virus, whereas the right contralateral muscles were injected with PBS. Muscles were analysed 2 and 6 months post‐injection to evaluate the short‐ and long‐term effects of *Sorbs1* exon 25 misregulation (Figure 2a).

**FIGURE 3:**
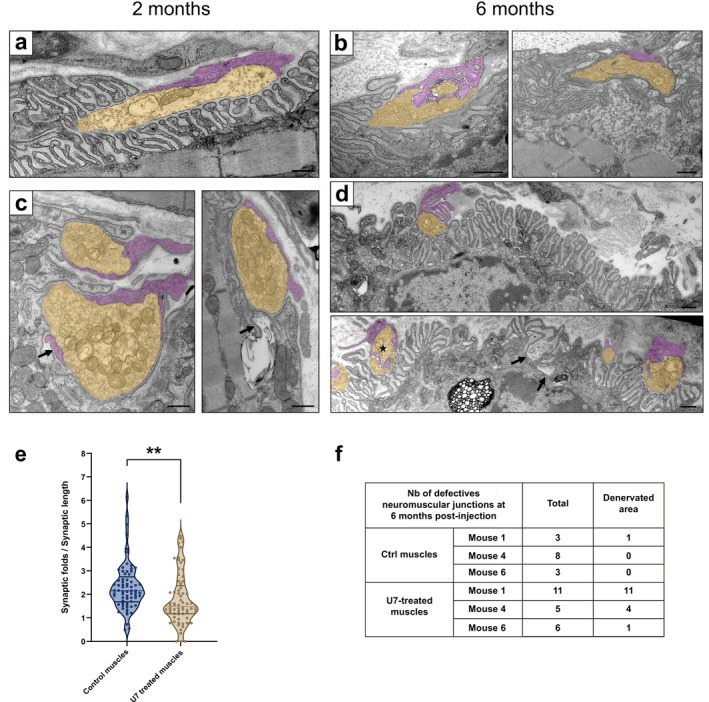
Exclusion of *Sorbs1* exon 25 in mice skeletal muscle disorganizes NMJ ultrastructural organization. Panels a and b are PBS‐treated tibialis anterior; panels c and d are U7‐ESE25–treated tibialis anterior. (a) Upper image is a NMJ from PBS‐treated tibialis at 2 months post injection. The upper part of the neuronal presynaptic bouton is recovered by a terminal Schwann cell process and faces numerous well‐ordered parallel post‐synaptic junctional folds. (c) NMJs from U7‐ESE25–treated tibialis at 2 months post injection. Presynaptic boutons appear normal with numerous synaptic vesicles and mitochondria, while there is a reduction of post‐junctional fold density with rare and short junctional folds. In the left image, a terminal Schwann cell process intrudes into the primary synaptic cleft (arrow), while in the right image, the arrow points a degenerative structure in the post‐synaptic zone. (d) NMJ from U7‐ESE25–treated tibialis at 6 months post injection. Marked endplate denervation with small presynaptic boutons facing only a small portion of the numerous postsynaptic folds. In the lower image, a neuronal bouton is invaded by terminal Schwann cell processes (asterisk). Arrows point to a group of disorganized junctional folds with an abnormal enlarged secondary cleft full of degenerative vesicles. Presynaptic terminals are pseudo‐coloured in yellow, and terminal Schwann cell are pseudo‐coloured in purple. For (a–d) scale bars = 500 nm. (e) Violin plots showing the number of post‐synaptic folds normalized to synaptic length in tibialis anterior (TA) muscles of control (left, blue) and U7‐SORBS1‐ESE25–injected (right, brown) mice, 2 months post‐injection. Each data point corresponds to a serial section of NMJs, with a total of 16 NMJs analysed. **p < 0.01, unpaired Student's *t*‐test. (f) Table providing a summary of the NMJ alterations observed in the muscles of 6‐month‐old mice used for electron microscopy.

Expression of *U7‐SORBS1‐ESE25* in TA mouse muscles reproduced the exon 25 mis‐splicing observed in DM1 embryonic muscle biopsies, leading to a reduction in exon inclusion of 70.4% ± 2.3% and 49.2% ± 8.1% at 2 and 6 months post‐injection, respectively (Figure 2b). This splicing alteration occurred without affecting the overall expression level of the SORBS1 transcript (Figure S3a). Despite these molecular alterations, no significant defects were observed in the injected TA muscles at 2 months post‐injection. Haematoxylin and eosin (H&E) staining of muscle tissue revealed no obvious structural abnormalities in muscle fibres, and immunofluorescence analysis of NMJ structure showed no significant changes in NMJ morphology and organization (Figures 2c,d and S3b). Nonetheless, transmission electron microscopy (TEM) analysis uncovered distinct structural abnormalities such as (i) a reduction in the number of post‐junctional folds, indicating compromised post‐synaptic membrane integrity, (ii) the invasion of the primary synaptic cleft by terminal Schwann cell processes and (iii) the presence of degenerative structures within the post‐synaptic compartment in TA muscles injected with *U7‐SORBS1‐ESE25* (Figure 3a,c).

By 6 months post‐injection, immunofluorescence analysis revealed a subset of NMJs with a pronounced reduction in innervation, as shown by a significant decrease in the mean nerve terminal area, based on the measurements from 327 NMJs in U7‐treated mice and 234 NMJs in control muscles (10.5% ± 3.4%; Figures 2e and S3c,d). In addition, further analysis demonstrated a significant reduction in the mean AChR area, indicating a progressive decline in NMJ integrity between 2 and 6 months post‐treatment (5.7% ± 2.5%; Figure 2f). These observations were corroborated by TEM analysis, which demonstrated pronounced structural impairments, including highly disorganized junctional folds with enlarged secondary clefts and numerous degenerative vesicles (Figure 3b,d). Additionally, ultrastructural analysis also revealed significant endplate denervation and smaller presynaptic boutons in treated TA muscles (Figure 3d). For reference, quantification of post‐synaptic folds in NMJs at 2 months post‐injection revealed a 22% ± 7.7% reduction in the number of folds normalized to synaptic length (Figure 3e). At 6 months post‐injection, the effect appeared more pronounced. In one mouse, all 11 NMJs analysed presented varying degrees of denervation, with some displaying markedly extended denervated regions. In another, four out of five NMJs exhibited regions of denervation, indicating widespread synaptic disruption. In the third mouse, one NMJ showed clear denervation, while three others displayed reduced junctional folding, suggestive of postsynaptic simplification or structural immaturity (Figure 3f). Interestingly, the sarcomere organization of TA muscles remained intact in both 2 and 6 months post‐injection, with no observed changes in muscle fibre ultrastructure (Figure S4a,b). While no molecular or structural alterations were observed at 2 months, RT‐qPCR analysis at 6 months revealed a significant downregulation of *Myh1* (encoding MyHC‐2X), along with a trend towards decreased expression of *Myh6* and *Myh7*, whereas *Myh2* (MyHC‐2A) and *Myh4* (MyHC‐2B) levels remained unchanged (Figure S4c). These molecular changes, observed only at the later time point, suggest a selective vulnerability of specific fast fibre subtypes and are consistent with a progressively evolving neuromuscular defect. These changes suggest that loss of the 56 amino acids encoded by the exon 25 of *SORBS1* may impair NMJ integrity and contribute to progressive neuromuscular alterations over time. However, as our experimental model does not allow us to clearly distinguish between defects in NMJ formation and maintenance, we cannot conclusively determine the precise stage at which this disruption occurs.

### 3.3 Exclusion of *sorbs1* Exon 25 During Zebrafish Development Impairs Locomotor Phenotype

**FIGURE 4:**
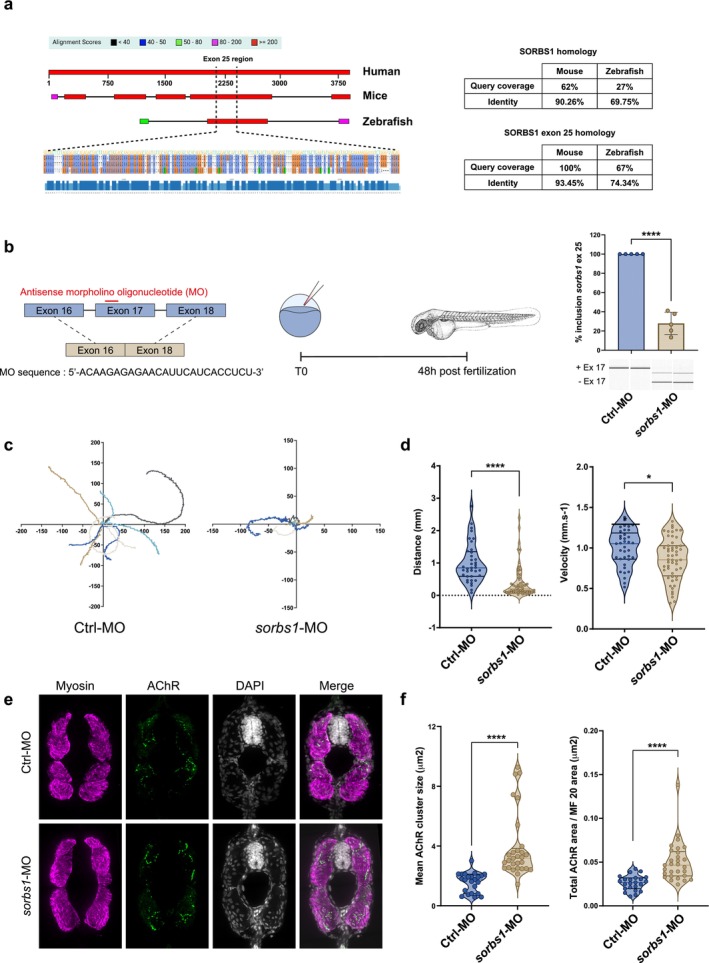
Exclusion of *sorbs1* exon 17 during zebrafish development impairs locomotor phenotype. (a) Sequence alignment and domain mapping of the *SORBS1* gene highlight a conserved region encompassing human exon 25, mouse exon 25 and zebrafish exon 17. This exon lies within the LIM domain‐containing region of the SORBS1 protein and exhibits high sequence conservation—with 93% identity between human and mice and 74% identity between human and zebrafish—underscoring its evolutionary conservation and functional relevance. Sequences from humans, mice and zebrafish have been aligned and compared using the Basic Local Alignment Search Tool (BLAST). (b) Schematic representation of the exon‐skipping strategy using antisense morpholino oligonucleotide (MO), and RT‐PCR analysis and quantification of sorbs1 exon 25 inclusion on total RNA extracts isolated from whole Ctrl‐MO and sorbs1‐MO embryos (48hpf); *p* < 0.0001, *N* = 5, unpaired Student's *t*‐test. Zebrafish eggs are microinjected with 0.6 mM of morpholino, and analysis is realized at 48 h post‐fertilization (hpf). (c) Colour‐coded video‐recorded tracks of swim trajectories of individual Ctrl‐MO and sorbs1‐MO embryos stimulated in touch‐evoked escape response (TEER) assay. (d) Violin plots representing the distribution of swimming distance and swimming velocity in TEER assay measured in cm, Ctrl‐MO (*N* = 37); sorbs1‐MO (*N* = 52) from three independent experiments *p* < 0.001 for distance, *p* < 0.05 for velocity, unpaired Student's *t*‐test. (e) Representative immunofluorescence of cryosectioned 48hpf zebrafish embryos. Images are confocal Z projections from Ctrl‐MO and sorbs1‐MO zebrafish. (f) Violin plots representing the distribution of AChR clusters size in mm^2^ and the distribution of AChR area normalized by skeletal muscle area in mm^2^; Ctrl‐MO (*n* = 23) from at least six fish; sorbs1‐MO (*n* = 29) from at least six fishes from three independent experiments. *p* < 0.001, unpaired Student's *t*‐test.

To investigate the consequences of *SORBS1* exon 25 exclusion during neuromuscular development, the exon‐skipping strategy was employed in zebrafish embryos (Figure 4b). The *sorbs1* gene in zebrafish consists of 25 exons, with exon 17 corresponding to exon 25 in the human gene. Notably, the orthologous exon in zebrafish (exon 17) shares 74% sequence identity with human exon 25, underscoring its evolutionary conservation and supporting the relevance of this alternative splicing event across species (Figure 4a). Expression profiling across key stages of zebrafish development (Figure S5a) revealed a significant upregulation of *sorbs1* transcripts containing exon 17 between 6 and 18 h post‐fertilization, coinciding with increased *rapsn* expression, a critical NMJ assembly gene. This developmentally regulated isoform switch supports the use of antisense morpholino oligonucleotides (AMOs) to block exon 17 inclusion during the critical window of NMJ formation. To prevent the developmental splicing switch of *sorbs1*, antisense morpholinos (MO) targeting the splicing site of *sorbs1* exon 17 were injected into one‐cell‐stage zebrafish embryos. PCR analysis at 50 h post‐fertilization (hpf) confirmed efficient exon 17 exclusion, resulting in a ΔPSI reduction of 72.1 ± 5.2% (Figure 4b). Because antisense morpholino injection in zebrafish embryos does not allow for tissue‐specific targeting, we note that despite effective exclusion of *sorbs1* exon 17, overall developmental abnormalities remained mild, especially compared to the pronounced motor phenotypes observed (see Figure S5b).

The swimming trajectory, distance and velocity were significantly reduced in sorbs1‐MO zebrafish compared to controls in the touch‐evoked escape response (TEER) test (57.9% ± 12% for distance and 14% ± 0.5% for velocity; Figure 4c,d, Figure S5c and Video S1). These findings demonstrate that exclusion of *sorbs1* exon 17 during neuromuscular development severely impairs locomotor function.

Immunofluorescence analysis of AChR cluster revealed alterations in NMJ organization (Figure 4e). In *sorbs1*‐MO larvae, AChR clusters appeared larger and aggregated in comparison to *mock‐injected larvae*, where AChR clusters were typically small and dispersed along the muscle fibres (Figure 4e). Quantitative analysis showed an increase in the mean size of individual AChR clusters (147% ± 29.7%; Figure 4f), as well as significant expansion in the total AChR area normalized to skeletal muscle area (92.3% ± 19.0%; Figure 4f). Examination of muscle tissues indicated that although the ‘U’ shaped myosepta remained intact in both control and *sorbs1*‐MO fishes, exon 17 exclusion caused a marked disruption in muscle fibre alignment (Figure S5d). This alteration was quantified by a reduction in proper fibre orientation, as measured by local anisotropy (24.32% *±* 5.4%; Figure S5e). In addition to its role in maintaining NMJ stability as observed in mice, these findings emphasize that the exclusion of *SORBS1* exon 25 during neuromuscular development results in motor impairments and disrupted AChR clustering.

### 3.4 SORBS1 Exon 25 Inclusion Is Mandatory to Generate Large Acetylcholine Receptor Clusters

**FIGURE 5:**
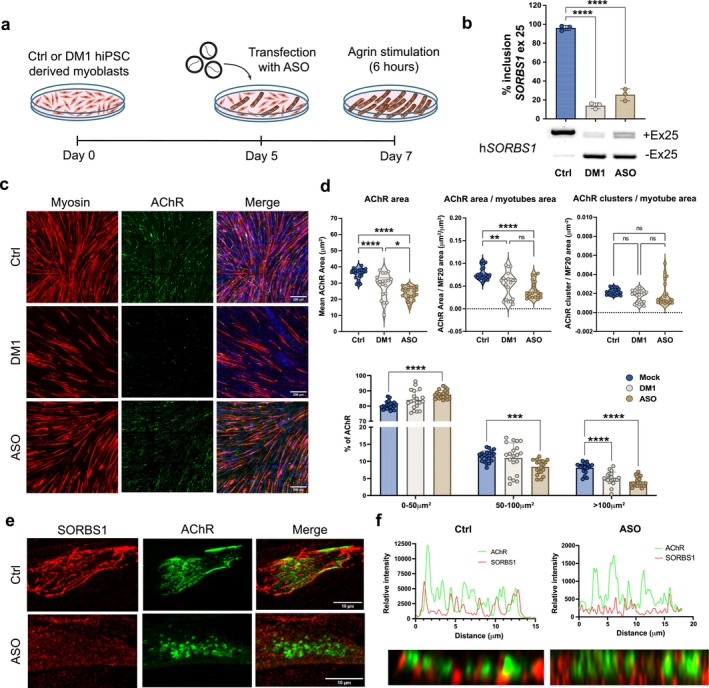
*SORBS1* correct splicing is mandatory to generate large AChR clusters. (a) Schematic representation of the exon‐skipping strategy using antisense oligonucleotide (ASO). hiPSCs‐derived myotubes from controls are terminally differentiated and transfected with 50 nM of ASO at 5 days. At Day 7 of differentiation, hiPSC‐derived myotubes are treated with 0.5 mg/mL of agrin for 6 h. (b) RT‐PCR analysis and quantification of *SORBS1* exon 25 inclusion on total RNA extracts isolated from Ctrl, DM1 and ASO‐transfected cell lysates. (c) Representative immunofluorescence of hiPSC‐derived myotubes at 7 days of differentiation and after 6 h of agrin stimulation (0.5 mg/mL). Images are confocal Z projections. (d) Violin plots representing the distribution of AChR clusters area in μm^2^; Ctrl (*n* = 21) from three independent experiments; DM1 (*n* = 21) from three independent experiments; ASO (*n* = 21) from three independent experiments. *p* < 0.01 and *p* < 0.0001, one‐way‐ANOVA followed by Tukey's post hoc test. Bar plots representing the distribution of AChR cluster sizes divided into groups of 0–50 mm^2^, 50–100 mm^2^ and superior to 100 mm^2^. *N* = 21 from three independent experiments. *p* < 0.001 and *p* < 0.0001, two‐way‐ANOVA. (e) Representative immunofluorescence of AChR clusters in Ctrl and ASO‐transfected hiPSC‐derived myotubes in high‐resolution confocal microscopy. (f) Fluorescence intensity profiles of AChR and SORBS1 in newly formed clusters following 6 h of agrin stimulation.

Because misregulation of *SORBS1* exon 25 disrupts NMJ formation in both mouse and zebrafish, we hypothesized that similar effects would occur in AChR clustering in human myotubes in response to agrin stimulation. To test this, hiPSC‐derived skeletal muscle cells were transfected with ASO sequence targeting the ESE25 region during the final stages of myogenic differentiation (Figure 5a), specifically on Day 5 when the myotube formation is initiated.

Transfection with ASO successfully reproduced the exon 25 mis‐splicing observed in hiPSC‐derived skeletal muscle cells from DM1 patients, leading to a ΔPSI reduction of 82.2% *±* 3.2% and 70.6% *±* 3.5% for DM1 and ASO, respectively (Figure 5b). Immunofluorescence analysis of AChR clusters at 7 days of differentiation, preceded by 6 h of stimulation with 0.5 μg/mL of agrin, revealed notable alterations in AChR cluster sizes (Figure 5c). Quantitative analysis showed a reduction of 34% *±* 4.5% in ASO‐treated hiPSC‐derived skeletal muscle cells (Figure 5d). Strikingly, a similar decrease in the mean size of AChR clusters was observed in DM1 hiPSC‐derived skeletal muscle cells (Figure 5d). Previous studies have demonstrated that skeletal myoblasts isolated from DM1 patients show reduced fusogenic capacity and diminished ability to form large and mature myotubes [11, 17]. To specifically isolate the effect of *SORBS1* exon 25 exclusion on AChR clustering, independent of fusogenic capacity, the total AChR area was normalized to the total myotube area. This analysis revealed reductions of 31.3% *±* 8.4% and 46.9% ± 8.3% in the total AChR area in DM1 and ASO‐treated skeletal muscle cells, respectively (Figure 5d). Interestingly, the total number of AChR clusters normalized to the total myotube area remained unchanged in both DM1 and ASO‐treated skeletal muscle cells (Figure 5d).

To further characterize the specific population of AChR clusters affected by exon 25 exclusion, the clusters were stratified into three size categories: 0–50 μm^2^, 50–100 μm^2^ and > 100 μm^2^ (Figure 5d). A significant reduction was observed in the number of very large AChR clusters (> 100 μm^2^) in both DM1 and ASO‐treated hiPSC‐derived skeletal muscle cells, suggesting that *SORBS1* exon 25 exclusion primarily disrupts AChR cluster size and organization rather than overall cluster formation. Specifically, the total number of clusters > 100 μm^2^ was reduced by 38.4% *±* 6.9% and 49.9% *±* 4.2% in DM1 and ASO‐treated cells, respectively (Figure 5d). To validate the effects of *SORBS1* exon 25 exclusion on the clustering of AChR in an independent model, we performed experiments in primary control skeletal muscle cells derived from a healthy individual (Figure S6a,b). These analyses confirmed a significant decrease in the total AChR cluster area normalized to myotubes area, with a reduction of 59.8% ± 18.9%, supporting the role of *SORBS1* exon 25 in regulating the clustering of AChR on the myotube membrane.

Using high‐resolution imaging with a LSM880 microscope equipped with an Airyscan module, we observed distinct spatial interdigitation between SORBS1 and AChR at the plasma membrane in control skeletal muscle cells (Figure 5e). Detailed examination revealed that SORBS1 localization at the plasma membrane was altered upon exon 25 exclusion, transitioning from large, packed protein clusters to more dispersed and fragmented clusters (Figure S6c). Notably, in ASO‐treated hiPSC‐derived skeletal muscle cells with mis‐spliced exon 25, the typical juxtaposition of SORBS1 and AChR clusters was disrupted, resulting in a shift from ‘plaque‐like’ AChR formations to more aggregated, irregular clusters (Figure 5e). Scanning the immunofluorescence intensity across the AChR clusters confirmed this interdigitation pattern in control cells, while ASO‐treated cells exhibited significantly reduced fluorescence intensity, indicating a reduction in the presence of protein at the membrane (Figure 5f). These findings indicate that *SORBS1* exon 25 mis‐splicing impairs the formation of large, organized AChR clusters in skeletal muscle cells, which may be critical for NMJ stability and proper neuromuscular function in DM1.

The observed reduction in AChR cluster size could result from either decreased insertion of new AChRs into the membrane or accelerated removal from existing clusters. To distinguish between these possibilities, we performed a dual‐labelling assay using α‐bungarotoxin (α‐BTX) conjugated to Alexa Fluor 488 and 647, allowing us to differentiate pre‐existing from newly inserted AChRs. As shown in Figure S6d, ASO‐treated myotubes displayed a significantly higher decrease in Alexa Fluor 488 labelled AChR, suggesting an impaired stability of AChR at post‐synaptic sites contributing to NMJ abnormalities observed.

To assess whether the reintroduction of SORBS1 exon 25 could restore the ability of DM1 cells to form mature AChR clusters, we overexpressed full‐length *SORBS1* mRNA via lentiviral transduction in DM1 hiPSC‐derived myoblasts (Figure S7a–c). In these experiments, forced expression of the full‐length transcript led to a modest increase in the mean AChR cluster size (Figure S7d,e), supporting a functional role for SORBS1 exon 25 in maintaining neuromuscular junction integrity.

## 4 Discussion

This study demonstrates that *SORBS1* exon 25 inclusion is essential for NMJ formation and stability in both animal models and human skeletal muscle cells. Notably, the alternative splicing of *SORBS1* exon 25 is dysregulated in skeletal muscles of DM1 patients, which may contribute to the neuromuscular impairments associated with the disease.

In skeletal muscle biopsies from human control fetuses, *SORBS1* exon 25 inclusion was first observed at 11 gestational weeks (Figure 1), which coincides with the formation of the first NMJs during human embryogenesis. This temporal correlation suggests a potential connection between the developmental regulation of *SORBS1* exon 25 inclusion and the establishment of neuromuscular connectivity. The inclusion of *SORBS1* exon 25 results in the addition of 54 amino acids, forming two α‐helices upstream of the protein's first SH3 domain. SORBS1, also known as ponsin or CAP, is an adaptor protein containing three SH3 domains that mediate interactions with various partners. Notably, SORBS1 binds Cbl, playing a critical role in the insulin signalling pathway [18, 19]. It localizes to cell‐extracellular matrix and cell–cell adhesion sites, where it interacts with proteins such as afadin, vinculin and paxillin [13, 20]. Depletion of SORBS1 in HEK293 cells resulted in cytoskeletal disorganization, increasing cell spreading ability associated with increased cell migration [12]. Beyond these roles, SORBS1 biological functions were also largely associated with cancers, as several SORBS1 mutations have been observed in different studies conducted on human cohorts [21, 22, 23].

In heart and skeletal muscles, SORBS1 has been described to colocalize with vinculin in costameres, where it facilitates the interaction between the cell membrane and filamin C [24, 25, 26]. SORBS1 appears to serve as a molecular bridge between the contractile actin cytoskeleton and membrane‐associated protein scaffolds. In mouse skeletal muscle cells, SORBS1 has been shown to interact with the CRK‐like protein (CRKL), a key player in promoting acetylcholine receptor (AChR) cluster formation, and its depletion disrupts AChR clustering following agrin stimulation [14]. Building on these findings, we explored whether the inclusion of exon 25 in SORBS1 could interfere with its interaction with CRKL and thereby contribute to the observed defects in AChR organization. Using immunoprecipitation assays, we confirmed that SORBS1 interacts robustly with CRKL; however, the presence of exon 25 does not appear to perturb this interaction (Figure S6f). These results suggest that the impaired AChR clustering associated with *SORBS1* exon 25 inclusion is unlikely to stem from disrupted CRKL binding and may instead involve other molecular mechanisms or downstream signalling pathways. Together, our findings expand the known functions of SORBS1, highlighting a novel role in the spatial organization and integrity of the neuromuscular synapse, beyond its established contribution to skeletal muscle structure.

Exon 25 of *SORBS1* is highly conserved across the different transcripts expressed in skeletal muscle tissues as well as in different species such as mice and zebrafish, underscoring a critical function for this coding sequence [27]. Our results demonstrate that increased skipping of *SORBS1* exon 25 leads to NMJ destabilization attested by the loss of post‐synaptic folds organization and consequently motoneuron denervation. Furthermore, the presence of Terminal Schwann cells within the synaptic clefts of some affected NMJ suggests a decrease in cellular membrane contact integrity due to the loss of exon 25 inclusion. However, although no major functional phenotype was observed in vivo, further studies involving sensitive muscle strength and electrophysiological testing may help clarify the functional relevance of the subtle structural changes induced by *SORBS1* exon 25 exclusion.

Forcing exclusion of the *SORBS1* orthologous exon 25 (exon 17) in zebrafish embryos led to strongly impaired locomotor phenotypes associated with disorganized AChR clusters. Notably, immunofluorescence analyses in zebrafish revealed enlarged AChR clusters, contrasting with findings in mouse and human skeletal muscle cell models. Although *SORBS1* exon 25 exhibits high sequence conservation across human, mouse and zebrafish, the zebrafish experiments primarily address the developmental consequences of exon 25 exclusion, rather than its role in mature neuromuscular function.

The larger AChR clusters observed in zebrafish may reflect compensatory mechanisms similar to those reported following the loss of agrin‐LRP4 signalling during zebrafish development [28]. The agrin signalling, mediated by the MUSK‐LRP4 receptor tyrosine kinase complex, plays a key role in AChR clustering [29, 30]. The downstream signalling cascade during the early minutes following agrin stimulation involves the activation of the Rac1 GTPase, PAK1 and actin cytoskeleton reorganization [29, 31]. Other signalling pathways such as GSK3β phosphorylation following AKT signalling have also been described as being implicated in CLASP2 stabilization at microtubule growing ends [32]. However, the protein interactions of SORBS1 and associated signalling, as well as the molecular consequences of its mis‐splicing, remain to be fully elucidated.

NMJ formation is tightly orchestrated by a cascade of signal exchanges between motoneurons and skeletal muscle fibres. Among the pathways involved, the Agrin/LRP4/MuSK/Dok7 signalling cascade is one of the best characterized mechanisms critical for NMJ development [33]. Mutations in these genes have been implicated in congenital neuromuscular disorders including congenital myasthenic syndrome (CMS), although most CMS cases result from mutations in AChR subunits [34]. In addition, some CMS cases arise from mutations affecting pre‐mRNA splicing [35], suggesting that splicing factors and their regulated alternative splicing events may play a critical role in NMJ formation and maintenance. For instance, the splicing factor SRSF1 has been implicated in NMJ development, as SRSF1‐deficient mice fail to form mature NMJs, likely due to impaired regulation of the alternative splicing of the CHRNE and AChR subunits [36].

Neuromuscular junction dysfunction has been documented in DM1, with neurophysiological studies across different patient cohorts identifying abnormalities in neuromuscular transmission [37, 38]. Despite this, the molecular mechanisms underlying these phenotypes remain poorly understood. The sequestration of MBNL protein by toxic (CUG)n‐repeats RNA leads to widespread splicing misregulation, potentially disrupting key NMJ‐associated splicing events. Notably, recent studies have shown that MBNL depletion in motor neurons impairs NMJ maintenance and function, although the precise mechanisms of MBNL action remain unclear [10, 39]. Our findings extend the contribution of MBNL proteins to the integrity of the NMJ at the post‐synaptic level. In alignment with this, the MBNL1 KO mouse model exhibits *SORBS1* exon 25 mis‐splicing along with significant NMJ morphological alterations [40].

The abnormalities observed in hiPSC‐derived skeletal muscle cells, including a reduced capacity to form large AChR clusters and a decrease in cluster stability, suggest that *SORBS1* splice defects contribute to compromised NMJ integrity in DM1. At the cellular level, our findings support a potential link between *SORBS1* exon 25 mis‐splicing and the ultrastructural defects observed at the NMJ in DM1 mouse models, pointing to a broader impact of toxic CUG repeat expansion and MBNL protein sequestration on neuromuscular synapse homeostasis.

In conclusion, this study highlights the critical role of MBNL‐dependent regulation of *SORBS1* exon 25 splicing in the formation of stable AChR clusters and the maintenance of ultrastructural NMJ integrity. By associating the misregulation of *SORBS1* exon 25 splicing with NMJ dysfunction, we provide insights into how splicing defects contribute to the neuromuscular impairments observed in DM1. These findings emphasize the broader impact of splicing dysregulation on NMJ homeostasis and the need to explore other splicing events in DM1. Additionally, this study stresses the importance of developing therapies to restore or preserve NMJ integrity. Finally, our results also point to the significance of understanding the interplay between splicing regulation and cytoskeletal organization at the NMJ, which could reveal additional therapeutic targets for stabilizing synaptic functions.

## Conflicts of Interest

The authors declare no conflicts of interest.

## Supporting information


**Figure S1:** (a) Bar graph of *SORBS1* exon 25 inclusion in hiPSC‐derived skeletal muscle cells from WT, DM1 and DKO MBNL1/2 (*N* = 4 independent experiments, ****p < 0.00001, one‐way ANOVA followed by Tukey's post hoc test). (b) Bar graph representing quantification of *SORBS1* exon 25 inclusion in skeletal muscle biopsies from human fetuses. *p* < 0.001, unpaired Student's *t*‐test. (c) Violin plots of total *SORBS1* mRNA in TA muscle samples from control and DM1 patients obtained from the publicly available DMseq database. Each dot represents an individual sample. (d) Correlation between the splice inclusion of *SORBS1* exon 25, *BIN1* exon 11 and *DMD* exon 78 inclusion in tibialis anterior muscles samples and the ankle dorsiflexion force (%) from the publicly available DMseq database. (e) Heatmap indicating the group hierarchy of *SORBS1* exon 25 inclusion from the tibialis anterior skeletal muscle samples according to their proximity. (f) Heatmap indicating the group hierarchy of *SORBS1* exon 25 inclusion from the heart muscle samples according to their proximity.
**Figure S2:** (a) Schematic representation of the different antisense oligonucleotide (ASO) tested and their targeted region on *SORBS1* pre‐mRNA. ISE, intron splice enhancer; ISS, intron splice suppressor; ESE, exon splice enhancer; ESS, exon splice suppressor; BP, branching point; SA, acceptor site; SD, donor site. (b) PCR experiments of *SORBS1* exon 25 splicing profile in human primary myotubes 48 h post‐transfection (*N* = 2 independent experiments). (c) RT‐PCR experiments of *SORBS1* mRNA containing the exon 25 in Ctrl, DM1 and Ctrl‐transfected hiPSC‐derived myotubes with the ASO‐ESE relative to Ctrl (*N* = 4 independent experiments, *p* < 0.0001, one‐way ANOVA followed by Tukey's post hoc test). (d) RT‐qPCR experiments of total *SORBS1* and total *SORBS2* mRNA in Ctrl, DM1 and Ctrl‐transfected hiPSC‐derived myotubes with the ASO‐ESE relative to Ctrl (*N* = 4 independent experiments, **p < 0.01, one‐way ANOVA followed by Tukey's post hoc test). (e) Representative Western blot of total SORBS1 and β‐ACTIN protein from Ctrl, DM1 and Ctrl‐transfected hiPSC‐derived myotubes with the ASO‐ESE. (f) Densitometric analysis of the Western blots. Quantification was performed on all detected bands by using Image J. The ratio of the relative level of SORBS1 to β‐ACTIN is plotted for each experimental condition (*N* = 4 independent experiments, one‐way ANOVA followed by Tukey's post hoc test).
**Figure S3:** (a) RT‐qPCR analysis and quantification of total *Sorbs1* and *Sorbs2* mRNA in TA muscles injected with AAV‐U7‐ESE25 compared with contralateral TA muscles injected with PBS (Ctrl) at 2 and 6 months (*N* = 5 mice at 2 months, and *N* = 6 mice at 6 months, one‐way ANOVA followed by Tukey's post hoc test). Data were normalized with the geometric mean between B2M, mRPLP0, TBP and PPIA mRNA levels and are presented as the mean ± SD value. (b) Haematoxylin and eosin staining in TA muscles injected either with PBS or with AAV‐U7‐SORBS1‐ESE25 at 2 months post‐injection. (c) Violin plots of presynaptic outputs using NMJ‐Morph at 6 months post‐injection. Ctrl TA (*n* = 234) from *N* = 6 mice; U7‐treated TA (*n* = 327) from *N* = 6 mice. Student's *t*‐test. (d) Violin plots of postsynaptic outputs using NMJ‐Morph (*N* = 5, *n* = 234) at 6 months post‐injection. Ctrl TA (*n* = 234) from *N* = 6 mice; U7‐treated TA (*n* = 327) from *N* = 6 mice. Student's *t*‐test.
**Figure S4:** (a) Sarcomeres just below NMJs from U7‐treated TA muscles at 2 months post injection. (b) Sarcomeres just below NMJs from U7‐treated TA muscles at 6 months post injection. Scale bars = 1000 nm. (c) Quantification by RT‐qPCR of total *Myh1*, *Myh2*, *Myh3*, *Myh4*, *Myh6* and *Myh7* mRNA in TA muscles injected with AAV‐U7‐ESE25 compared with contralateral TA muscles injected with PBS (Ctrl) at 2 and 6 months (*N* = 5 mice at 2 months, and *N* = 6 mice at 6 months, **p < 0.01, one‐way ANOVA followed by Tukey's post hoc test). Data were normalized with the geometric mean between B2M, mRPLP0, TBP and PPIA mRNA levels and are presented as the mean ± SD value.
**Figure S5:** (a) RT‐qPCR quantification of transcript levels for *sorbs1* containing exon 17, total *sorbs1* containing exon 5, total *sorbs2*, *musk* and *rapsn*. (*N* ≥ 2 independent experiment with *n* = 30 fish per experiment). Data were normalized with *Eif1* mRNA levels and are presented as the mean ± SD value. (b) Stacked bar plot showing the proportion of embryos presenting normal morphology, motor phenotype, developmental abnormalities or death at 48 hpf in three experimental groups: non‐injected (NI, *n* = 20), mismatch morpholino control (Mis, *n* = 28) and splice‐blocking morpholino targeting *sorbs1* exon 17 (*sorbs1*‐MO, *n* = 24). Only viable fish without developmental aberrations have been kept for analysis. (c) Time lapse from recorded videos of swimming comportment of individual Ctrl‐MO and *sorbs1*‐MO embryos stimulated in touch‐evoked escape response (TEER) assay. (d) Representative immunofluorescence of cryosectioned 48hpf zebrafish embryos. Images are confocal Z projections from Ctrl‐MO and *sorbs1*‐MO zebrafish. Muscle fibre orientations are colour coded using Orientation J plugin in ImageJ. (e) Violin plots representing distribution of muscle fibre anisotropy; Ctrl‐MO (*n* = 23) from at least six fish; *sorbs1*‐MO (*n* = 29) from at least six fish. *p* < 0.0001, unpaired Student's *t*‐test.
**Figure S6:** (a) Representative immunofluorescence of primary human myotubes at 3 days of differentiation and after 6 h of agrin stimulation (0.5 mg/mL). Images are confocal Z projections. (b) Violin plots representing the distribution of AChR clusters area in mm^2^; Ctrl (*n* = 7) from two independent experiments; ASO (*n* = 7) from two independent experiments. *p* < 0.01, unpaired Student's *t*‐test. (c) Representative immunofluorescence of SORBS1 location on the membrane of primary human myotubes at 3 days of differentiation. (d) Schematic of the experimental timeline (top): Control hiPSC‐derived myotubes were transfected with ASO on Day 5, stimulated with agrin (500 ng/mL) on Day 7 for 6 h and sequentially labelled with α‐bungarotoxin (α‐BTX) conjugated to Alexa Fluor 488 (to label pre‐existing AChRs) and Alexa Fluor 647 (to label total AChRs after 24 h of agrin stimulation). Representative confocal images (bottom left) show α‐BTX 488 (green), α‐BTX 647 (red) and merged channels in control (Ctrl) and ASO‐treated myotubes. Scale bars: 10 μm. Quantification (bottom right) of the area of AChR clusters labelled with α‐BTX 488 at 24 h, quantification of the area of AChR clusters labelled with α‐BTX 647 at 24 h and percentage of remaining AChR clusters labelled with α‐BTX 488 to AChR clusters labelled with α‐BTX 647 at 24 h (**p < 0.01, *p < 0.05 unpaired Student's *t*‐test). (e) RT‐qPCR analysis and quantification of total *MUSK* and *RAPSN* mRNA in control, DM1 and ASO‐treated myotubes (at least three independent experiments, *p < 0.05, one‐way ANOVA followed by Tukey's post hoc test). Data were normalized with the 18S mRNA levels and are presented as the mean ± SD value. (f) Western blots of the co‐immunoprecipitation between CRKL and SORBS1 in Ctrl, DM1 and ASO‐treated myotubes. Cell lysates in native conditions containing 100 μg of proteins were subjected to immunoprecipitation with anti‐CRKL antibodies. Bound proteins were detected with specific antibodies against CRKL or SORBS1.
**Figure S7:** (a) Schematic representation of the exon *SORBS1* rescue strategy using lentivirus infection. hiPSCs‐derived myotubes from DM1 patients are infected for 24 h at Day 2 and allowed to recover for an additional 24 h. The cells are then terminally differentiated into myotubes for 7 days. At Day 7 of differentiation, hiPSC‐derived myotubes are treated with 0.5 mg/mL of agrin for 6 h. (b) RT‐PCR analysis and quantification of SORBS1 exon 25 inclusion on total RNA extracts isolated from non‐infected (NI) and infected DM1 cells with multiplicity of infection (MOIs) of 0.5, 1 and 5. (c) Representative images of DM1 hiPSC‐derived myoblasts infected with different MOI of GFP lentivirus. Images have been taken 24 h post‐infection. (d) Representative immunofluorescence of hiPSC‐derived myotubes at 7 days of differentiation and after 6 h of agrin stimulation (0.5 mg/mL). Images are confocal Z projections. (e) Violin plots representing the distribution of AChR clusters area in μm^2^ and the number of AChR clusters rationalized by MF20 area (μm^−2^); NI (*n* = 44) from two independent experiments; MOI 1 (*n* = 36) from two independent experiments. *p < 0.05, unpaired Student's *t*‐test.

**Table S1:** Supporting Information.
