# Supplementary material for: Alternative Splicing of SORBS1 Affects Neuromuscular Junction Integrity in Myotonic Dystrophy Type 1
Source: J Cachexia Sarcopenia Muscle. 2025 Nov 18;16(6):e70112. doi: 10.1002/jcsm.70112 (PMC12624223; doi:10.1002/jcsm.70112)
Supplement: Supplementary file 1 — Figure S1: (a) Bar graph of SORBS1 exon 25 inclusion in hiPSC‐derived skeletal muscle cells from WT, DM1 and DKO MBNL1/2 (N = 4 independent experiments, ****p < 0.00001, one‐way ANOVA followed by Tukey's post hoc test). (b) Bar graph representing quantification of SORBS1 exon 25 inclusion in skeletal muscle biopsies from human fetuses. p < 0.001, unpaired Student's t‐test. (c) Violin plots of total SORBS1 mRNA in TA muscle samples from control and DM1 patients obtained from the publicly available DMseq database. Each dot represents an individual sample. (d) Correlation between the splice inclusion of SORBS1 exon 25, BIN1 exon 11 and DMD exon 78 inclusion in tibialis anterior muscles samples and the ankle dorsiflexion force (%) from the publicly available DMseq database. (e) Heatmap indicating the group hierarchy of SORBS1 exon 25 inclusion from the tibialis anterior skeletal muscle samples according to their proximity. (f) Heatmap indicating the group hierarchy of SORBS1 exon 25 inclusion from the heart muscle samples according to their proximity. Figure S2: (a) Schematic representation of the different antisense oligonucleotide (ASO) tested and their targeted region on SORBS1 pre‐mRNA. ISE, intron splice enhancer; ISS, intron splice suppressor; ESE, exon splice enhancer; ESS, exon splice suppressor; BP, branching point; SA, acceptor site; SD, donor site. (b) PCR experiments of SORBS1 exon 25 splicing profile in human primary myotubes 48 h post‐transfection (N = 2 independent experiments). (c) RT‐PCR experiments of SORBS1 mRNA containing the exon 25 in Ctrl, DM1 and Ctrl‐transfected hiPSC‐derived myotubes with the ASO‐ESE relative to Ctrl (N = 4 independent experiments, p < 0.0001, one‐way ANOVA followed by Tukey's post hoc test). (d) RT‐qPCR experiments of total SORBS1 and total SORBS2 mRNA in Ctrl, DM1 and Ctrl‐transfected hiPSC‐derived myotubes with the ASO‐ESE relative to Ctrl (N = 4 independent experiments, **p < 0.01, one‐way ANOVA followed by Tukey's post hoc [file JCSM-16-e70112-s001.docx]

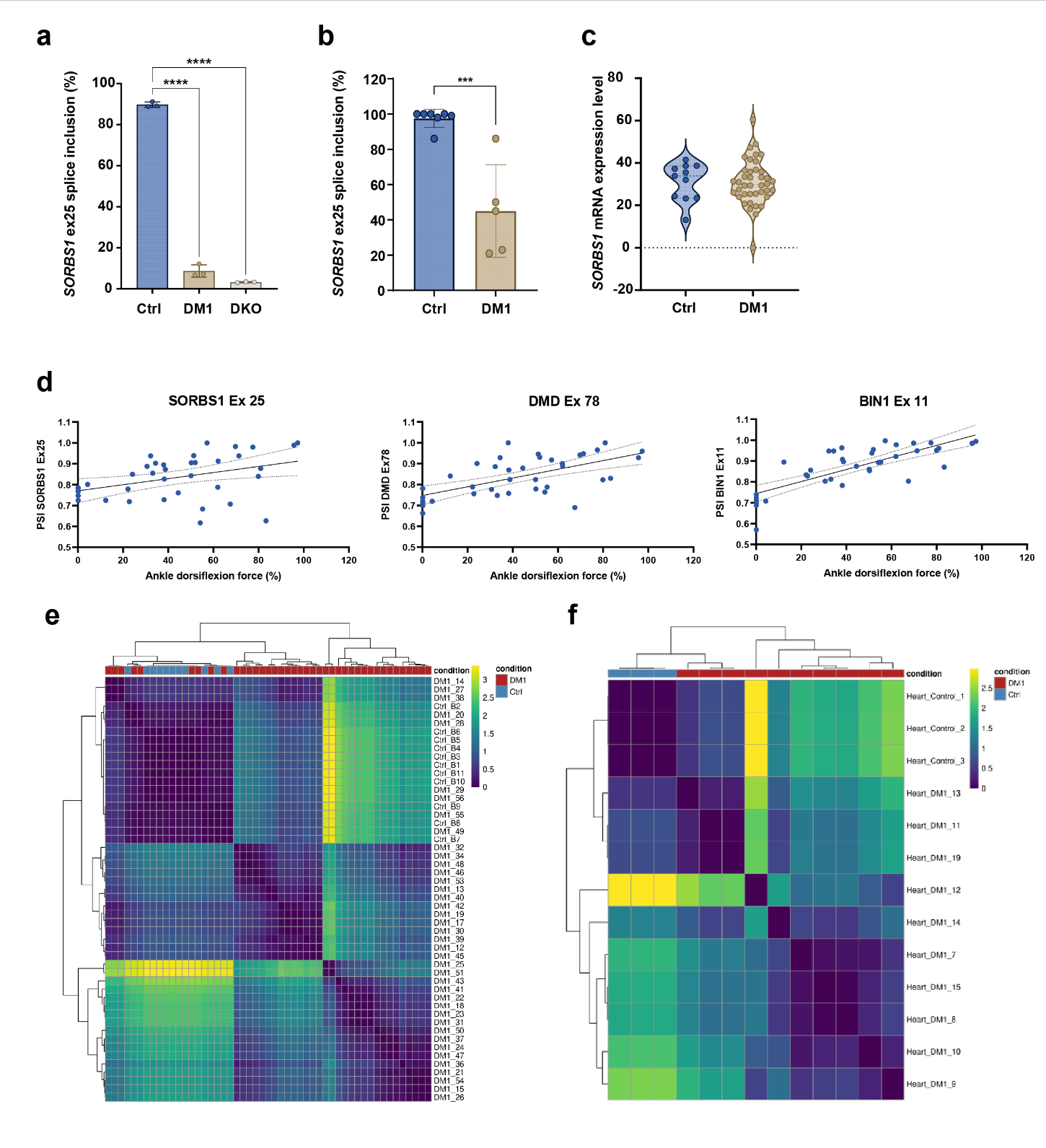
**SUPPLEMENTARY FIGURES**

**Supplementary figure 1** I **(a)** Bar graph of SORBS1 exon 25 inclusion in hiPSC-derived skeletal muscle cells from WT, DM1 and DKO MBNL1/2 (N =4 independent experiments, **** p < 0.00001, one-way ANOVA followed by Tukey’s post hoc test). **(b)** Bar graph representing quantification of SORBS1 exon 25 inclusion in skeletal muscle biopsies from human fetuses. p < 0.001, unpaired Student’s t-test. (**c**) Violin plots of total SORBS1 mRNA in TA muscle samples from control and DM1 patients obtained from the publicly available DMseq database. Each dot represents an individual sample. (**d**) Correlation between the splice inclusion of *SORBS1* exon 25, *BIN1* exon 11, and *DMD* exon 78 inclusion in tibialis anterior muscles samples and the ankle dorsiflexion force (%) from the publicly available DMseq database. (**e**) Heatmap indicating the group hierarchy of SORBS1 exon 25 inclusion from the tibialis anterior skeletal muscle samples according to their proximity. (**f**) Heatmap indicating the group hierarchy of SORBS1 exon 25 inclusion from the heart muscle samples according to their proximity.


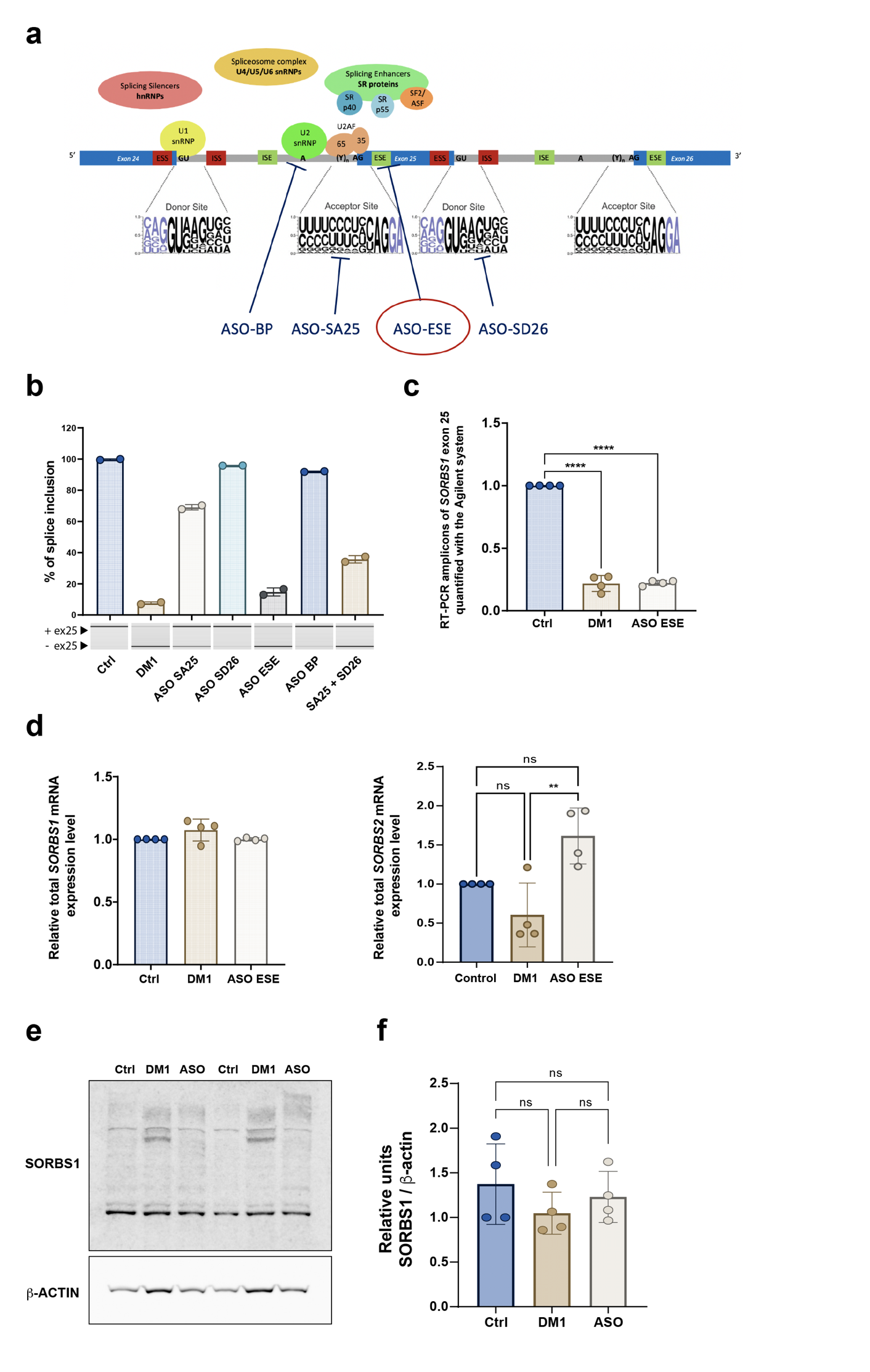


**Supplementary figure 2** I (**a**) Schematic representation of the different antisense oligonucleotide (ASO) tested and their targeted region on *SORBS1* pre-mRNA. ISE: Intron Splice Enhancer, ISS: Intron Splice Suppressor, ESE: Exon Splice Enhancer, ESS: Exon Splice Suppressor, BP: Branching Point, SA: Acceptor Site, SD: Donor Site (**b**) PCR experiments of SORBS1 exon 25 splicing profile in human primary myotubes 48h post transfection (N=2 independent experiments). (**c**) RT-PCR experiments of SORBS1 mRNA containing the exon 25 in Ctrl, DM1 and Ctrl-transfected hiPSC derived myotubes with the ASO-ESE relative to Ctrl. (N = 4 independent experiments, p < 0.0001, one-way ANOVA followed by Tukey’s post hoc test). (**d**) RT-qPCR experiments of total SORBS1 and total SORBS2 mRNA in Ctrl, DM1 and Ctrl-transfected hiPSC derived myotubes with the ASO-ESE relative to Ctrl. (N = 4 independent experiments, ** p < 0.01, one-way ANOVA followed by Tukey’s post hoc test). (**e**) Representative Western blot of total SORBS1 and β-ACTIN protein from Ctrl, DM1, and Ctrl-transfected hiPSC derived myotubes with the ASO-ESE. (**f**) Densitometric analysis of the western blots. Quantification was performed on all detected bands by using Image J. The ratio of the relative level of SORBS1 to β-ACTIN is plotted for each experimental condition (N = 4 independent experiments, one-way ANOVA followed by Tukey’s post hoc test).


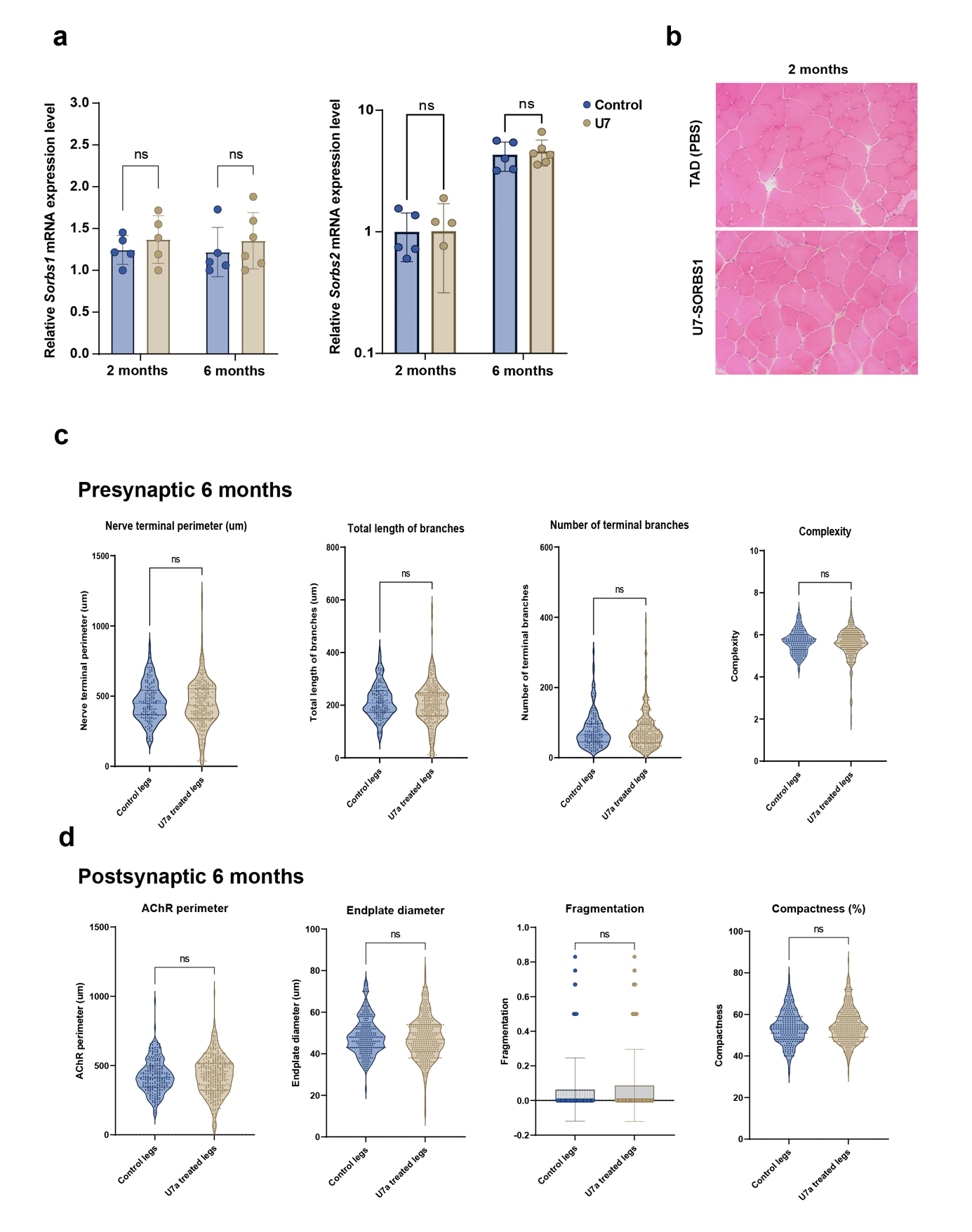


**Supplementary figure 3** I (**a**) RT-qPCR analysis and quantification of total *Sorbs1* and *Sorbs2* mRNA in TA muscles injected with AAV-U7-ESE25 compared with contralateral TA muscles injected with PBS (Ctrl) at 2-months and 6-months (N = 5 mice at 2 months, and N = 6 mice at 6 months, one-way ANOVA followed by Tukey’s post hoc test). Data were normalized with the geometric mean between B2M, mRPLP0, TBP and PPIA mRNA levels and are presented as the mean ± SD value. (**b**) Hematoxylin and eosin staining in TA muscles injected either with PBS or AAV-U7-SORBS1-ESE25 at 2 months post-injection. (**c**) Violin plots of presynaptic outputs using NMJ-Morph at 6 months post-injection. Ctrl TA (n=234) from N = 6 mice; U7 treated TA (n=327) from N = 6 mice. Student’s t-test. (**d**) Violin plots of postsynaptic outputs using NMJ-Morph (N=5, n=234) at 6 months post-injection. Ctrl TA (n=234) from N = 6 mice; U7 treated TA (n=327) from N = 6 mice. Student’s t-test.


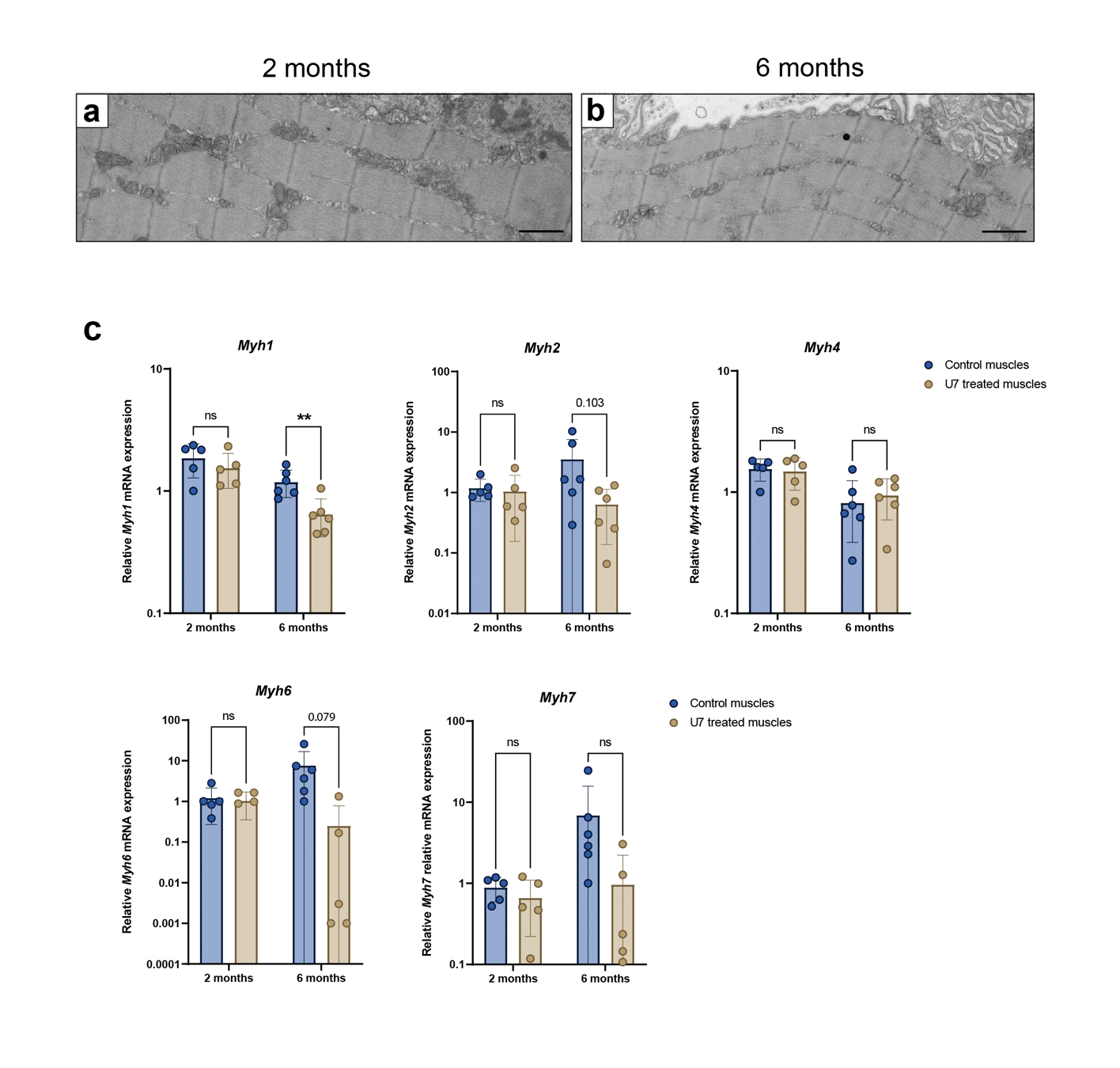


**Supplementary figure 4** I **(a)** Sarcomeres just below NMJs from U7 treated TA muscles at 2 months post injection. (**b**) Sarcomeres just below NMJs from U7 treated TA muscles at 6 months post injection. Scale bars= 1000 nm. (**c**) Quantification by RT-qPCR of total *Myh1, Myh2, Myh3, Myh4, Myh6,* and *Myh7* mRNA in TA muscles injected with AAV-U7-ESE25 compared with contralateral TA muscles injected with PBS (Ctrl) at 2-months and 6-months (N = 5 mice at 2 months, and N = 6 mice at 6 months, ** p < 0.01, one-way ANOVA followed by Tukey’s post hoc test). Data were normalized with the geometric mean between B2M, mRPLP0, TBP and PPIA mRNA levels and are presented as the mean ± SD value.


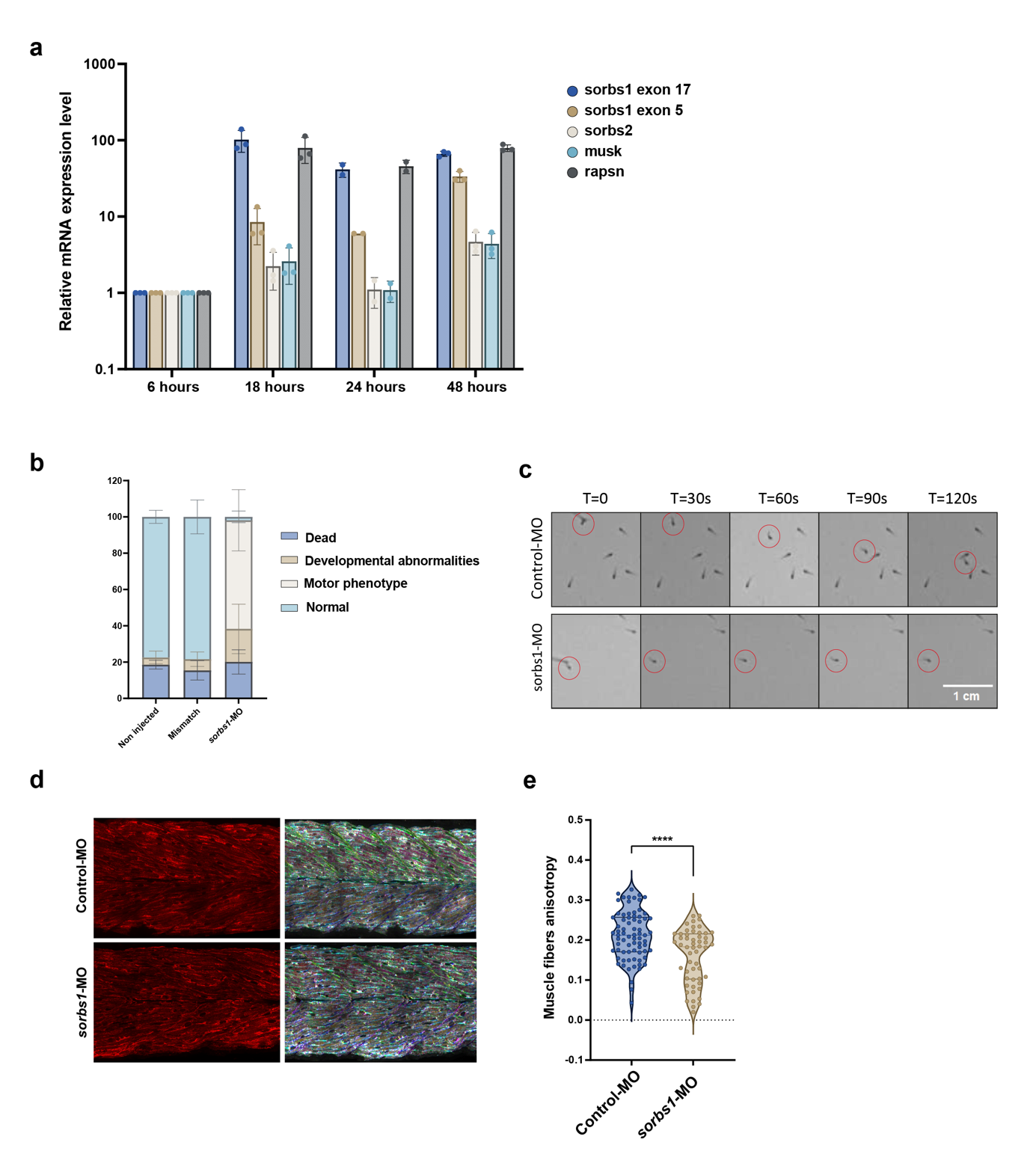


**Supplementary figure 5** I (**a**) RT-qPCR quantification of transcript levels for *sorbs1* containing exon 17, total *sorbs1* containing exon 5, total *sorbs2*, *musk*, and *rapsn*. (N≥2 independent experiment with n = 30 fish per experiment). Data were normalized with *Eif1* mRNA levels and are presented as the mean ± SD value. (**b**) Stacked bar plot showing the proportion of embryos presenting normal morphology, motor phenotype, developmental abnormalities, or death at 48 hpf in three experimental groups: non-injected (NI, n = 20), mismatch morpholino control (Mis, n = 28), and splice-blocking morpholino targeting *sorbs1* exon 17 (*sorbs1*-MO, n = 24). Only viable fish without developmental aberrations have been kept for analysis. (**c**) Time lapse from recorded videos of swimming comportment of individual Ctrl-MO and *sorbs1*-MO embryos stimulated in touch-evoked escape response (TEER) assay. (**d**) Representative immunofluorescence of cryosectioned 48hpf zebrafish embryos. Images are confocal Z projections from Ctrl-MO and *sorbs1*-MO zebrafish. Muscle fibers orientations are color coded using Orientation J plugin in ImageJ. (**e**) Violin plots representing distribution of muscle fibers anisotropy; Ctrl-MO (n=23) from at least 6 fish; *sorbs1*-MO (n=29) from at least 6 fish. P < 0.0001, unpaired Student’s t-test.


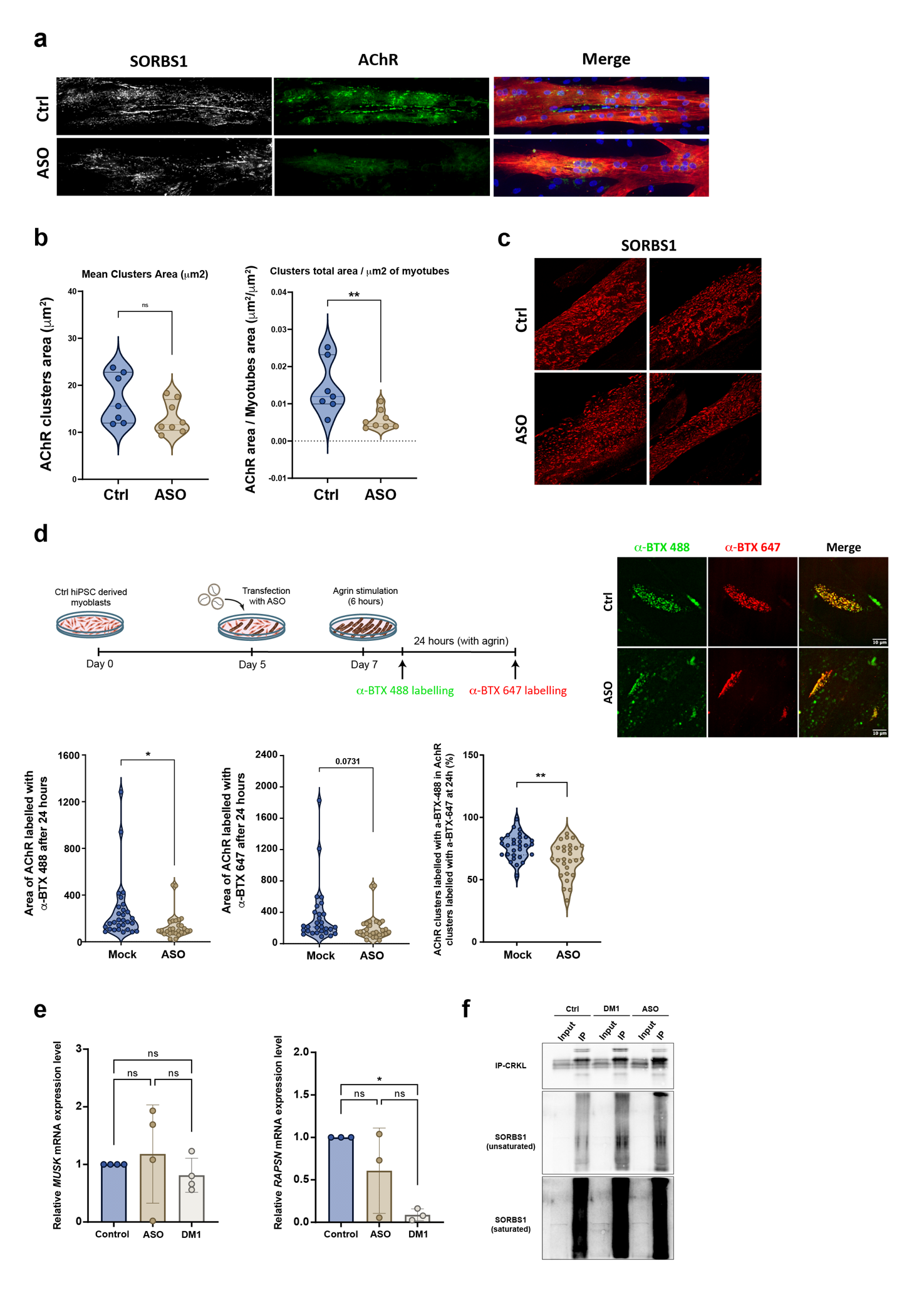


**Supplementary figure 6** I **(a)** Representative immunofluorescence of primary human myotubes at 3 days of differentiation and after 6 hours of agrin stimulation (0,5 mg/ml). Images are confocal Z projections. (**b**) Violin plots representing distribution of AChR clusters area in mm^2^; Ctrl (n=7) from 2 independent experiments; ASO (n=7) from 2 independent experiments. p < 0.01, unpaired Student’s t-test. **(c)** Representative immunofluorescence of SORBS1 location on the membrane of primary human myotubes at 3 days of differentiation. (**d**) Schematic of the experimental timeline (top): control hiPSC-derived myotubes were transfected with ASO on day 5, stimulated with agrin (500ng/ml) on day 7 for 6 hours, and sequentially labelled with α-bungarotoxin (α-BTX) conjugated to Alexa Fluor 488 (to label pre-existing AChRs) and Alexa Fluor 647 (to label total AChRs after 24 hours of agrin stimulation). Representative confocal images (bottom left) show α-BTX 488 (green), α-BTX 647 (red), and merged channels in control (Ctrl) and ASO-treated myotubes. Scale bars: 10 µm. Quantification (bottom right) of the area of AChR clusters labelled with α-BTX 488 at 24 hours, quantification of the area of AChR clusters labelled with α-BTX 647 at 24 hours, and percentage of remaining AChR clusters labelled with α-BTX 488 to AChR clusters labelled with α-BTX 647 at 24 hours (** p < 0.01, * p<0.05 unpaired Student’s t-test) (**e**) RT-qPCR analysis and quantification of total *MUSK and RAPSN* mRNA in control, DM1, and ASO-treated myotubes. (at least 3 independent experiments, * p < 0.05, one-way ANOVA followed by Tukey’s post hoc test). Data were normalized with the 18S mRNA levels and are presented as the mean ± SD value. (**f**) Western blots of the co-immunoprecipitation between CRKL and SORBS1 in Ctrl, DM1, and ASO-treated myotubes. Cell lysates in native conditions containing 100 μg of proteins were subjected to immunoprecipitation with anti-CRKL antibodies. Bound proteins were detected with specific antibodies against CRKL or SORBS1.


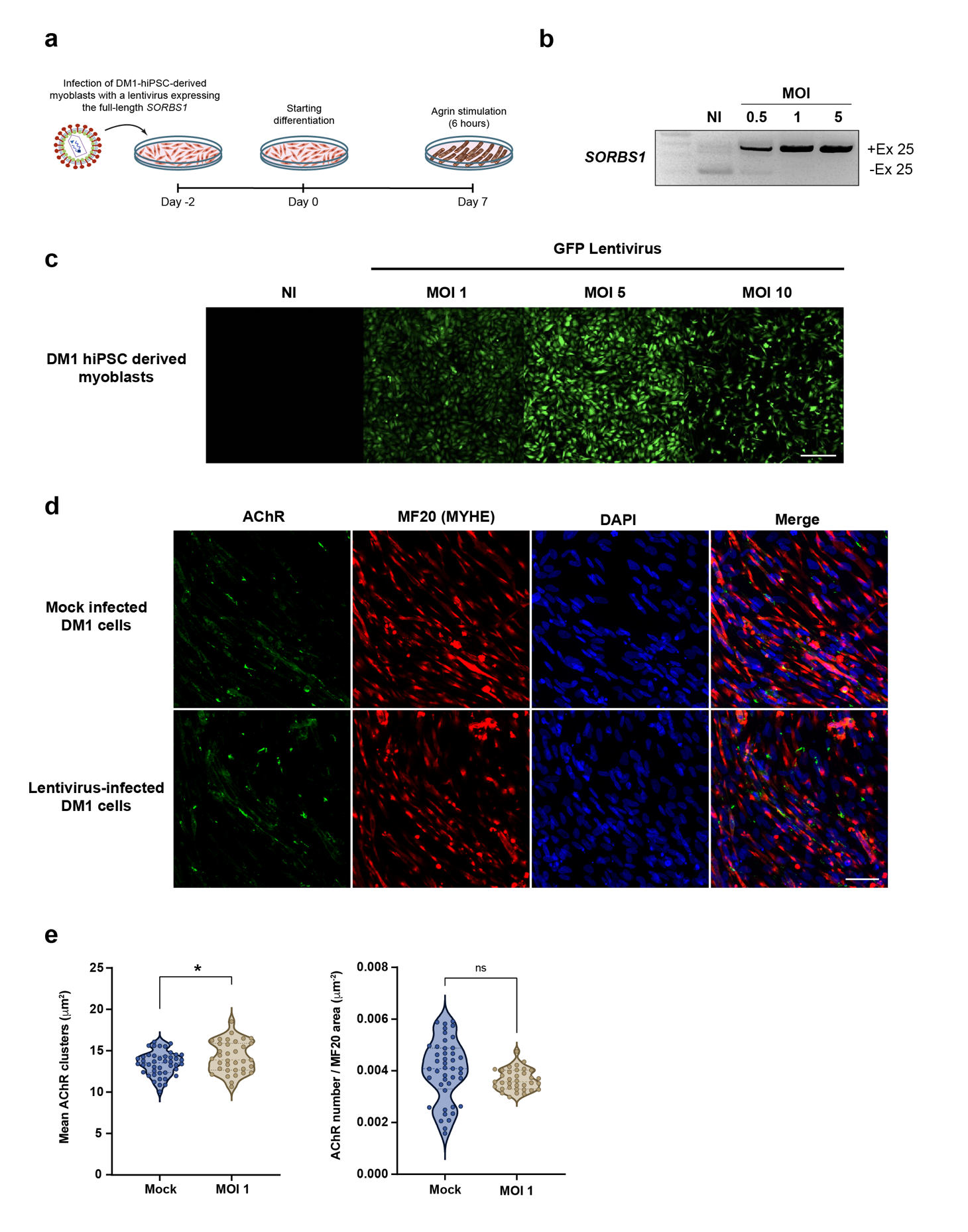


**Supplementary figure 7** I **(a)** Schematic representation of the exon *SORBS1* rescue strategy using lentivirus infection. hiPSCs derived myotubes from DM1 patients are infected for 24 hours at day 2 and allowed to recover for an additional 24 hours. The cells are then terminally differentiated into myotubes for 7 days. At day 7 of differentiation, hiPSC-derived myotubes are treated with 0.5 mg/ml of agrin for 6 hours. (**b**) RT-PCR analysis and quantification of SORBS1 exon 25 inclusion on total RNA extracts isolated from non-infected (NI) and infected DM1 cells with multiplicity of infection (MOIs) of 0.5, 1 and 5. (**c**) Representative images of DM1 hiPSC-derived myoblasts infected with different MOI of GFP lentivirus. Images have been taken 24 hours post-infection. (**d**) Representative immunofluorescence of hiPSC derived myotubes at 7 days of differentiation and after 6 hours of agrin stimulation (0.5 mg/ml). Images are confocal Z projections. (**e**) Violin plots representing distribution of AChR clusters area in μm^2^ and the number of AChR clusters rationalized by MF20 area (μm^-2^); NI (n=44) from 2 independent experiments; MOI 1 (n=36) from 2 independent experiments. * p < 0.05, unpaired Student’s t-test.
